# Color changing object recognition and grabbing technology based on crystal butterfly algorithm and adaptive imitation

**DOI:** 10.1016/j.isci.2024.110457

**Published:** 2024-07-06

**Authors:** Zuoxun Wang, Chuanyu Cui, Jinxue Sui, Changkun Guo

**Affiliations:** 1 School of Information and Electronic Engineering, Shandong Technology and Business University, No. 191, Binhai Middle Road, Laishan District, Yantai 264005, China

**Keywords:** Applied sciences, Algorithms

## Abstract

Implementing grasping tasks under color and multi scene promotion conditions is a key technology. This study proposes a recognition and grasping technique based on the crystal butterfly algorithm and adaptive imitation synthesis. Firstly, inspired by the movement trajectory of butterflies, a dynamic node tracking method called "Butterfly Trajectory" was designed. It can complete dynamic trajectory tracking under geometric constraints and achieve route memory. The second color dynamic recognition technology (CDR) has been proposed. It can quickly extract brightness, transparency, and color saturation obtained from multiple angles. Improve the feature extraction speed of Region CNN (R-CNN) instead of traditional methods (HOG). In addition, an adaptive imitation synthesis technique (AISP) is used to achieve the multi scenario promotion of grasping technology. Finally, simulation and physical testing were provided to verify the effectiveness of the design scheme in this article.

## Highlights

• Crystal Butterfly tracks dynamic color-changing nodes, enhancing data richness
• CDR in Crystal Butterfly improves dynamic color recognition via path memory
• AISP enhances grasping adaptability in complex environments

## Graphical abstract

**Figure:**
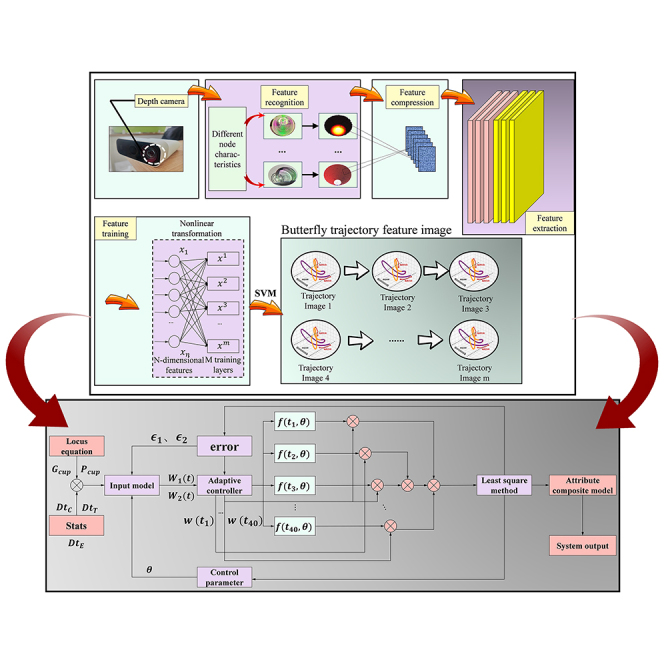

## Introduction

In recent years, due to the increase in cargo transportation and the shortage of human resources, people have become increasingly interested in the research of gripping technology. It is an attractive solution to use visual or tactile recognition technology^1^^,^^2^^,^^3^ as a "perception organ" to perform grasping tasks. This scheme has been widely verified in multiple fields.^4^^,^^5^^,^^6^^,^^7^ This technology also has broad application scenarios in modern medicine. Such as medical rehabilitation robots,^8^ automatic medicine-grabbing robots, and so forth.

The first article to employ recognition techniques in grasping technology seems to be [9].^9^ Since then, people have been very interested in studying recognition techniques to accomplish grasping tasks. Hay and Chehadeh^10^ proposed a noise-tolerant method based on a deep neural network (DNN). This method overcomes the rich domain knowledge required for system adjustment and can achieve real-time identification. Wang and Qiu^11^ studied a transformer based on CNN architecture. Used to solve visual place recognition problems. The R-CNN designed in^12^ uses the traditional CNN network instead of HOG to effectively improve the speed of feature extraction. It can quickly identify items to be grabbed among distractions. With the popularity of sensors, using them as identification mechanisms in grabbing has become another research area for scholars. Such as visual sensors,^13^ proximity sensors,^14^ and tactile sensors,^15^ and so forth. In order to fuse and extract different recognition information obtained during grasping, Weng and Zhou^16^^,^^17^ developed multi-modal datasets and multi-light sensing methods. They can provide a more comprehensive dataset for scraping. Along with the application of human-computer interaction, Li and Yin^18^ proposed a visual and tactile fusion framework. This method improves the grasping success rate in complex environments by complementing visual and tactile data. In order to increase the application scenarios of,^18^ Li S and Yu H^19^ proposed a transparent object grasping technology based on the fusion of vision and touch. However, the promotion of this technology in complex background environments still needs to be improved. Jiang^20^ designed a transparent object deep reconstruction and manipulation framework (A4T). This framework builds a large dataset (TRANS-AFF) to save object data and complete the detection of transparent objects.

It should be emphasized that most of the above model designs on recognition grasping focus on linear systems with fixed colors. Although^20^ considered the effect of transparent objects due to light reflection and refraction, it did not address the change of background color. [19]^19^ focuses in color change, but the promotion of complex scenes is poor. It can be seen that the recognition capture of color changing objects brings new challenges to the existing techniques. Therefore it is imperative to study the recognition grasping of color changing objects.

Based on the above observations, we investigate a recognition grasping technique based on the Crystal Butterfly algorithm and adaptive imitation synthesis. The contribution of this article is 3-fold.

1) A dynamic node tracking method called "butterfly trajectory" is proposed in the Crystal Butterfly algorithm. It can accurately track the trajectory of color-changing object nodes and change the dynamic node dataset in real time. The trajectory detection and tracking effect is better than [21],^21^ Compared with Dex-Nerf^22^ and RGB-D,^23^ it has richer data information.
2) A color dynamic recognition technology (CDR) is proposed in the Crystal Butterfly algorithm. To overcome the recognition bias caused by color changes,^20^^,^^21^^,^^22^^,^^23^^,^^24^ this method incorporates the path memory of the butterfly trajectory. This makes it easier to extract multi-angle features of color-changing objects. Compared with [12]^12^ and [19],^19^ it has higher dynamic color recognition.
3) To expand the application of recognition and grasping in complex environments, we proposed the adaptive imitation synthesis technology (AISP). Specifically, this method overcomes the shortcomings of imitation learning,^25^ such as poor data adaptability and simple copying, and focuses more on the adaptability of learning. It can continuously make new adjustments according to changes in environmental information and achieve multi-scenario grasping promotion.

The remainder of this article is organized as follows. Section II discusses the design process of the methodology regarding the dynamic node tracking of butterfly trajectories. Section III establishes the color dynamic recognition technique. Section IV discusses the establishment and application of adaptive mimicry synthesis. Section V gives simulations and physical tests to illustrate the theoretical results. Section VI summarizes this article. Section VII points out the limitations of the article and future research directions.

### Butterfly trajectory dynamic node tracking

The set of internal hierarchical nodes of a transparent object is represented as $I_{\text{cup}}$. This set contains *N* nodes. $I_{\text{cup}}=\left\{\left(x_{\text{in}1},y_{\text{in}1},z_{\text{in}1}\right),\left(x_{\text{in}2},y_{\text{in}2},z_{\text{in}2}\right),...,\left(x_{\text{inN}},y_{\text{inN}},z_{\text{inN}}\right)\right\}$. The set of grabbing points when grabbing the glass is $G_{\text{cup}}$. It indicates where the grab point should be placed. $G_{\text{cup}}=\left\{\left(x_{\text{grip}1},y_{\text{grip}1},z_{\text{grip}1}\right),\left(x_{\text{grip}2},y_{\text{grip}2},z_{\text{grip}2}\right)\right\}$. The center position of the transparent object is $P_{\text{cup}}=\left\{x_{\text{cup}},y_{\text{cup}},z_{\text{cup}}\right\}$. Both $G_{\text{cup}}$ and $P_{\text{cup}}$ are node collections at the external level. The internal hierarchical nodes and external hierarchical nodes are explained as follows.

1) Internal hierarchy nodes are key points of the internal geometry of transparent objects.
2) External hierarchy nodes are key points on the surface and edges of transparent objects.

These nodes are used to establish geometric relationships between each other. This allows the grasping device to adjust its posture based on the real-time position of the transparent object.

Select 5 key master nodes, represented by $A_{1},A_{2},A_{3},A_{4}$ and $A_{5}$ respectively. Since the position of a node is a function of time. Therefore $I_{\text{cup}}\left(t\right)=\{\;\left(\;x_{\text{in}1}\left(t\right),y_{\text{in}1}\left(t\right),{\;z}_{\text{in}1}\left(t\right)\right),\left(x_{\text{in}2}\left(t\right),y_{\text{in}2}\left(t\right),z_{\text{in}2}\left(t\right)),...,\left(x_{\text{inN}}\left(t\right),y_{\text{inN}}\left(t\right),z_{\text{inN}}\left(t\right)\;)\;\}.\;G_{\text{cup}}\left(t\right)=\{\left(x_{\text{grip}1}\left(t\right),y_{\text{grip}1}\left(t\right),z_{\text{grip}1}\left(t\right)\right),x_{\text{grip}1}\left(t\right),y_{\text{grip}1}\left(t\right),z_{\text{grip}1}\left(t\right)\right),\left(x_{\text{grip}1}\left(t\right),y_{\text{grip}1}\left(t\right),z_{\text{grip}1}\left(t\right)\right),x_{\text{grip}1}\left(t\right),y_{\text{grip}1}\left(t\right),z_{\text{grip}1}\left(t\right)\right),\left(x_{\text{inN}}\left(t\right),y_{\text{inN}}\left(t\right),z_{\text{inN}}\left(t\right)\;)\;\}.\;G_{\text{cup}}\left(t\right)=\{\left(x_{\text{grip}1}\left(t\right),y_{\text{grip}1}\left(t\right),z_{\text{grip}1}\left(t\right)\right),x_{\text{grip}1}\left(t\right),y_{\text{grip}1}\left(t\right),z_{\text{grip}1}\left(t\right)\right),x_{\text{inN}}\left(t\right),y_{\text{inN}}\left(t\right),z_{\text{inN}}\left(t\right)\;)\;\}.\;G_{\text{cup}}\left(t\right)=\{(x_{\text{grip}2}\left(t\right),y_{\text{grip}2}\left(t\right)$, $z_{\text{grip}2}\left(t\right))\}.\;P_{\text{cup}}\left(t\right)=\{x_{\text{cup}}\left(t\right),y_{\text{cup}}\left(t\right),z_{\text{cup}}\left(t\right)\}$.

**Figure 1:**
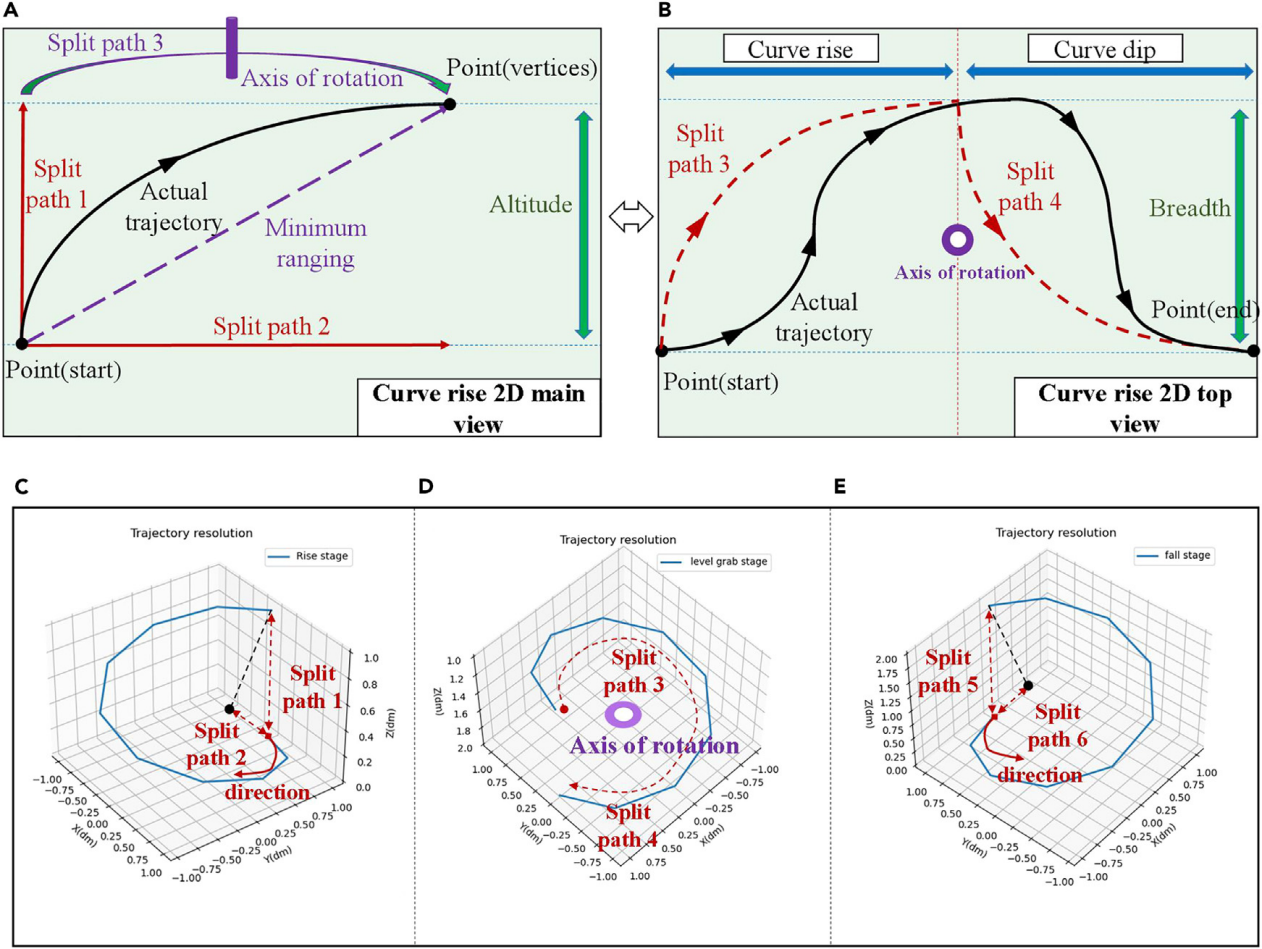
Trajectory split (A) is the main view trajectory route. (B) is the top view trajectory route. (C–E) is the trajectory route and separation path at different grabbing stages.

All the grabbing processes will go through grabbing (depalletizing), curve rising, curve descending and placing (palletizing). Therefore $G_{\text{cup}}$ will change from the initial position before grabbing to the final position. ${\;G}_{\text{cup}}\left(t_{0}\right)=\{\left(x_{\text{grip}1}\left(t_{0}\right),y_{\text{grip}1}\left(t_{0}\right),z_{\text{grip}1}\left(t_{0}\right)\right),\left({\;x}_{\text{grip}2}\left(t_{0}\right),y_{\text{grip}2}\left(t_{0}\right),z_{\text{grip}2}\left(t_{0}\right)\right)\}\;\to {\;G}_{\text{cup}}\left(t_{2}\right)=\{\left(x_{\text{grip}1}\left(t_{2}\right),y_{\text{grip}1}\left(t_{2}\right),z_{\text{grip}1}\left(t_{2}\right)\right),\left(x_{\text{grip}2}\left(t_{2}\right),y_{\text{grip}2}\left(t_{2}\right),z_{\text{grip}2}\left(t_{2}\right)\right)\}$. $t_{0}$ represents the start time of grasping, and $t_{2}$ represents the end time of grasping. The grasping trajectory is divided into different stages, as shown in Figure 1.

1) Rising stage.

The center point ($P_{\text{cup}}$) of the transparent object changes as it rises. $P_{\text{cup}}\left(t\right)=\{x_{\text{cup}}\left(t\right),y_{\text{cup}}\left(t\right),z_{\text{cup}}\left(t\right)\}$. $z_{\text{cup}}\left(t\right)$ will increase.

2) Horizontal rotation stage.

The position and direction of the grab point set ($G_{\text{cup}}$) change at the same time. ${\;G}_{\text{cup}}\left(t\right)=\left\{\left(x_{\text{grip}1}\left(t\right),{\;y}_{\text{grip}1}\left(t\right),z_{\text{grip}1}\left(t\right)\right),\left(x_{\text{grip}2}\left(t\right),{\;y}_{\text{grip}2}\left(t\right),z_{\text{grip}2}\;\left(t\right)\right)\right\}.\;x_{\text{grip}1}\left(t\right)$ and $x_{\text{grip}2}\left(t\right)$ will change.

3) Descending stage.

The grab point sets $G_{\text{cup}}$ and $P_{\text{cup}}$ change with descent. $G_{\text{cup}}\left(t\right)=\left\{\left(x_{\text{grip}1}\left(t\right),y_{\text{grip}1}\left(t\right),z_{\text{grip}1}\left(t\right)\right),\left(x_{\text{grip}2}\left(t\right),y_{\text{grip}2}\left(t\right),z_{\text{grip}2}\left(t\right)\right)\right\},P_{\text{cup}}\left(t\right)=\left\{x_{\text{cup}}\left(t\right),y_{\text{cup}}\left(t\right),z_{\text{cup}}\left(t\right)\right\}$. $z_{\text{grip}1}\left(t\right)$, $z_{\text{grip}2}\left(t\right)$ and $z_{\text{cup}}\left(t\right)$ will decrease.

Based on the above information, the trajectory equation of $G_{\text{cup}}$ is,

(Equation 1) $$G_{cup}\left(t\right)=\left\{ \begin{array}{cc} \left(x_{start1},y_{start1},z_{start1}+v_{up1}\left(t-t_{0}\right)\right), & t_{0}<t<t_{1}\\ \left(x_{rotatel1}\left(t-t_{1}\right),y_{rotatel1}\left(t-t_{1}\right),z_{rotatel1}\left(t-t_{1}\right)\right), & t_{1}<t<t_{2}\\ \left(x_{down1},y_{down1},z_{down1}-v_{down1}\left(t-t_{2}\right)\right), & t>t_{2} \end{array}\right.$$

where $t_{0},t_{1}$ and $t_{2}$ represent the time points when the grabbing occurs, the rotation occurs and the end time respectively. $x_{\text{start}1},{\;y}_{\text{start}1},z_{\text{start}1},x_{\text{rotatel}1},y_{\text{rotatel}1},z_{\text{rotatel}1},{\;x}_{\text{down}1},y_{\text{down}1},z_{\text{down}1}$ respectively represent the coordinate positions of three time nodes. $v_{\text{up}1}$ and $v_{\text{down}1}$ represent the rising and falling speeds.

The trajectory equations of $P_{\text{cup}}\left(t\right)$ and ${\;I}_{\text{cup}}\left(t\right)$ both satisfy,

(Equation 2) $$P_{cup}\left(t\right)=\left\{ \begin{array}{cc} \left(x_{start2}+f\left(t\right),y_{start2},z_{start2}\right), & {\;t}_{0}<t<t_{1}\\ (x_{rotatel2}+f\left(t_{1}\right)\cos\left(g\left(t\right)\right),y_{rotatel2}+f\left(t_{1}\right)sin\left(g\left(t\right),z_{rotatel2}\right)\; & t_{1}<t<t_{2}\\ (x_{down2}+f\left(t_{1}\right)\cos\left(g\left(t_{2}\right)\right)+h\left(t\right),y_{down2}+f\left(t_{1}\right)\sin\left(g\left(t_{2}\right)\right), & t>t_{2} \end{array}\right.$$

where $x_{\text{start}2},y_{\text{start}2},z_{\text{start}2},x_{\text{rotatel}2},y_{\text{rotatel}2},z_{\text{rotatel}2},x_{\text{down}2},y_{\text{down}2},z_{\text{down}2}$ respectively represent the coordinate positions of three time nodes. f(t) is the rising trajectory function. g(t) is the descending trajectory function. h(t) is the rotation function. $f\left(t_{1}\right)$ and $g\left(t_{2}\right)$ are the rising trajectory function and falling trajectory function at $t_{1}$ and ${\;t}_{2}$ moments, respectively. The motion trajectory of the glass grabbing point satisfies (1). The motion trajectories of the five key nodes all satisfy (2). Consider picking an arbitrary point from keypoints and grab points. Take $A_{1}$ as an example. Trajectory tracking via trajectory equations. Complete route memory for transparent objects. Assume ${\;A}_{1}=\left(x,y,z\right)$, establish a second-order motion model,

(Equation 3) $$A_{1}\left(t\right)={[x\left(t\right),y\left(t\right),z\left(t\right),\dot{x}\left(t\right),\dot{y}\left(t\right),\dot{z}\left(t\right),\ddot{x}\left(t\right),\ddot{y}\left(t\right),\ddot{z}\left(t\right)]}^{T}$$

$A_{1}\left(t\right)$ is the status vector. This formula contains the position information, velocity information, and acceleration information during the movement of the node. According to the second-order motion model, the state transition equation is,

(Equation 4) $$A_{1}\left(t\right)=BA_{1}\left(t-1\right)+\text{CU}\left(t\right)+\delta_{t}$$

where B is the state transition matrix. C is the control input matrix. U(t) is the control input vector. $\delta_{t}$ is the process noise in the state transition equation.

If the different stages of trajectory splitting are regarded as uniform linear motion, then the state transition matrix at this time can be expressed as,

(Equation 5) $$B=\left[\begin{array}{ccccccccc} 1 & 0 & 0 & \Delta t & 0 & 0 & \frac{1}{2}{\left(\Delta t\right)}^{2} & 0 & 0\\ 0 & 1 & 0 & 0 & \Delta t & 0 & 0 & \frac{1}{2}{\left(\Delta t\right)}^{2} & 0\\ 0 & 0 & 1 & 0 & 0 & \Delta t & 0 & 0 & \frac{1}{2}{\left(\Delta t\right)}^{2}\\ 0 & 0 & 0 & 1 & 0 & 0 & \Delta t & 0 & 0\\ 0 & 0 & 0 & 0 & 1 & 0 & 0 & \Delta t & 0\\ 0 & 0 & 0 & 0 & 0 & 1 & 0 & 0 & \Delta t\\ 0 & 0 & 0 & 0 & 0 & 0 & 1 & 0 & 0\\ 0 & 0 & 0 & 0 & 0 & 0 & 0 & 1 & 0\\ 0 & 0 & 0 & 0 & 0 & 0 & 0 & 0 & 1 \end{array}\right]$$

where Δt is the time interval between adjacent stages in the grasping process.

The control input matrix is expressed as,

(Equation 6) $$C=\left[\;\begin{array}{ccc} 0 & 0 & 0\\ 0 & 0 & 0\\ 0 & 0 & 0\\ 1 & 0 & 0\\ 0 & 1 & 0\\ 0 & 0 & 1\\ 0 & 0 & 0\\ 0 & 0 & 0\\ 0 & 0 & 0 \end{array}\;\right]$$

This control matrix can realize speed control of ${\;A}_{1}$ in the three directions of $\dot{x}\left(t\right)$, $\dot{y}\left(t\right)$ and $\dot{z}\left(t\right)$ during constant speed rise, horizontal constant speed rotation, and constant speed descent. However, there is an actual curved motion during the grabbing process. Its trajectory direction and velocity magnitude are constantly changing. Therefore, the state transition matrix and control input matrix for the curve motion trajectory become,

(Equation 7) $$B=\left[\begin{array}{ccccccccc} 1 & 0 & \Delta t & 0 & \frac{1}{2}{\left(\Delta t\right)}^{2} & 0 & 0 & 0 & 0\\ 0 & 1 & 0 & \Delta t & 0 & \frac{1}{2}{\left(\Delta t\right)}^{2} & 0 & 0 & 0\\ 0 & 0 & 1 & 0 & \Delta t & 0 & \frac{1}{2}{\left(\Delta t\right)}^{2} & 0 & 0\\ 0 & 0 & 0 & 1 & 0 & \Delta t & 0 & \frac{1}{2}{\left(\Delta t\right)}^{2} & 0\\ 0 & 0 & 0 & 0 & 1 & 0 & \Delta t & 0 & \frac{1}{2}{\left(\Delta t\right)}^{2}\\ 0 & 0 & 0 & 0 & 0 & 1 & 0 & \Delta t & 0\\ 0 & 0 & 0 & 0 & 0 & 0 & 1 & 0 & \Delta t\\ 0 & 0 & 0 & 0 & 0 & 0 & 0 & 1 & 0\\ 0 & 0 & 0 & 0 & 0 & 0 & 0 & 0 & 1 \end{array}\right]$$

(Equation 8) $$C=\left[\;\begin{array}{ccc} 0 & 0 & 0\\ 0 & 0 & 0\\ 0 & 0 & 0\\ 0 & 0 & 0\\ 1 & 0 & 0\\ 0 & 1 & 0\\ 0 & 0 & 1\\ 0 & 0 & 0\\ 0 & 0 & 0 \end{array}\;\right]$$

During the trajectory tracking process, the observation equation is set to,

(Equation 9) $$O\left(t\right)=PA_{1}\left(t\right)+\varepsilon_{t}$$

(Equation 10) $$P=\left[\begin{array}{ccccccccc} 1 & 0 & 0 & 0 & 0 & 0 & 0 & 0 & 0\\ 0 & 1 & 0 & 0 & 0 & 0 & 0 & 0 & 0\\ 0 & 0 & 1 & 0 & 0 & 0 & 0 & 0 & 0 \end{array}\right]$$

where P is the observation matrix. $\varepsilon_{t}$ is the observation noise. O(t) is the observation vector.

During the observation process, the state transition equation and observation equation are updated as,

(Equation 11) $$\hat{A_{1}}\left(t\right)=B\hat{A_{1}}\left(t-1\right)+\text{CU}\left(t\right)+\delta_{t}$$

(Equation 12) $$\hat{O}\left(t\right)=P\hat{A_{1}}\left(t\right)+\varepsilon_{t}$$

where $\hat{A_{1}}\left(t\right)$ is the updated state transition function at time t. $\hat{A_{1}}\left(t-1\right)$ is the state transition function at time t-1 before the update. $\hat{O}\left(t\right)$ is the updated observation function at time t.

According to the trajectory equation motion model, the state transition equation and the observation equation are estimated and predicted.

(Equation 13) $$\hat{A_{1}}\left(t\right)=B\hat{A_{1}}\left(t-1\right)+{\;G}_{\text{cup}}\left(t\right)\left[\text{PCU}\left(t\right)+\varepsilon_{t}\right]$$

(Equation 14) $$\hat{O}\left(t\right)=P\hat{A_{1}}\left(t\right)+{\;P}_{\text{cup}}\left(t\right)\left[\text{PCU}\left(t\right)+\delta_{t}\;\right]$$

**Figure 2:**
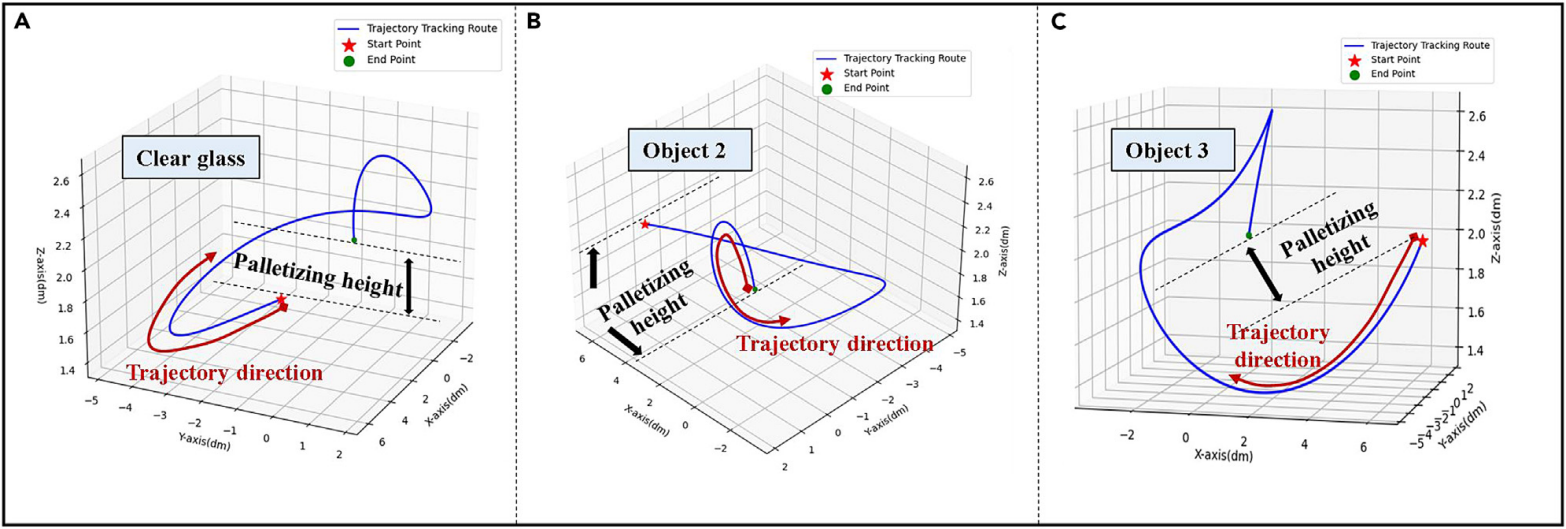
The trajectory of different items (A) is the trajectory of a transparent glass. (B) is the trajectory of a non-transparent object. (C) is the trajectory of another non-transparent object.

**Figure 3:**
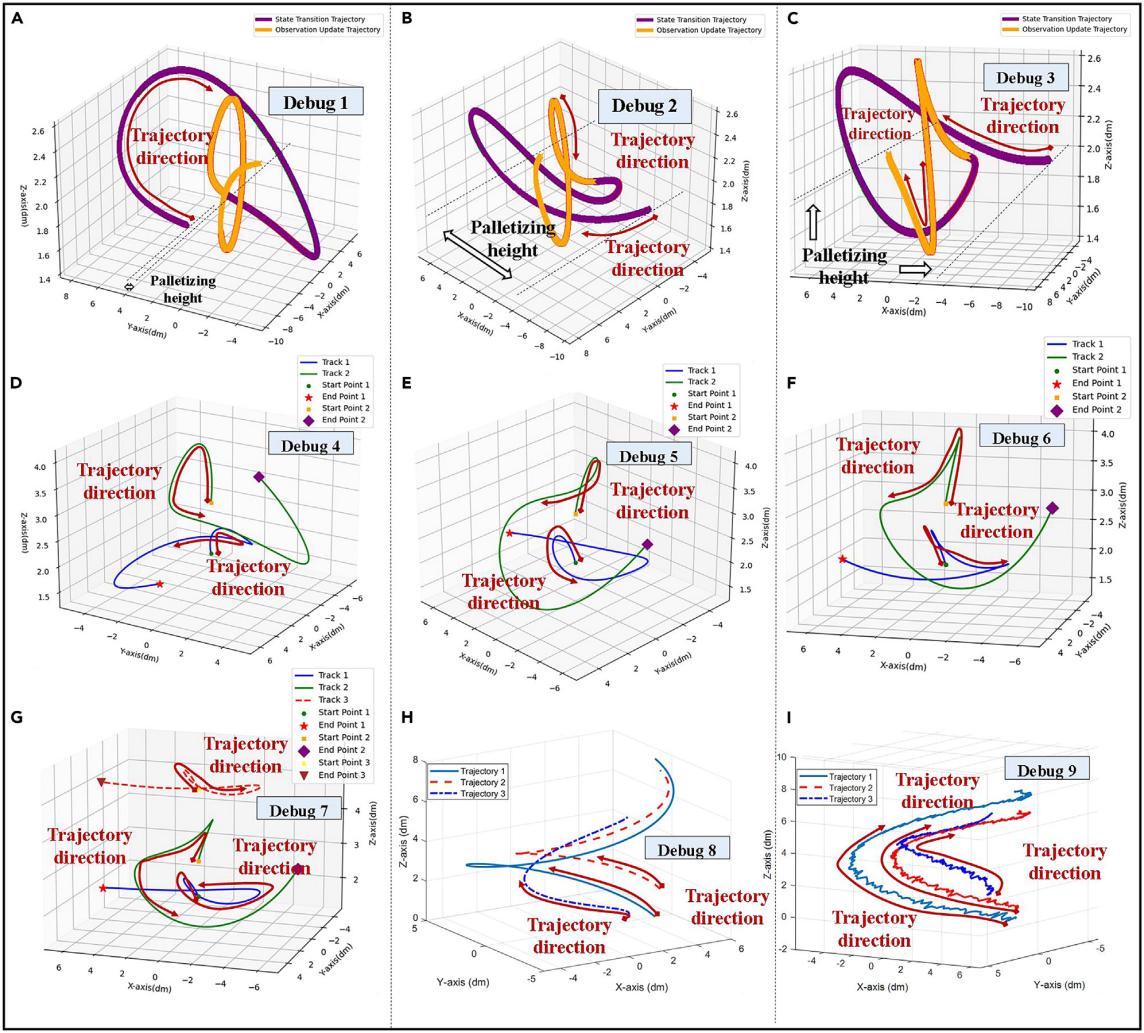
Dynamic debugging of trajectory route (A–C) is the dynamic trajectory of one key node. (D–F) is the dynamic trajectory of two key nodes. (G–I) is the dynamic trajectory of three key nodes.

Based on the above observations, Figure 2 shows the trajectories of different objects. Figure 3 is the dynamic debugging process for different numbers of trajectory routes in the update of state transition equations and observation equations. It can be seen from Figures 2 and 3 that the stacking height and trajectory direction of different objects are different. Therefore, the trajectory of each object is different.

**Remark 1:** The butterfly trajectory dynamic node tracking method uses the second-order motion model and state transition matrix to describe the motion process of transparent objects. In this method, the internal hierarchical node set and the external hierarchical node set of the transparent object are set, and these nodes represent the key feature points of the object. The grasping trajectory is split into different stages. The trajectory equations and observation equations of different nodes collect rich motion data compared to [22–23].^22^^,^^23^ The estimated prediction of the state transition equation combined with the control input can be used to perform the dynamic trajectory tracking of key nodes in different stages. It is worth noting that the dynamic node tracking method is only suitable for grasping tasks of transparent items of specific shapes. It is not suitable when the grabbing environment is interfered with by non-transparent items with the same shape and different colors as the grabbing target. Therefore, it is necessary to establish color dynamic recognition technology (CDR) based on this method.

### Color dynamic recognition technology (CDR)

CDR abandons the feature extraction method of traditional R-CNN.^12^ Its key ingredients are the fusion of multimodal data into a comprehensive dataset and diversified feature analysis.

According to the dynamic trajectory, n action photos of transparent objects are captured through the depth camera. The depth camera model used in the experimental test is Kinect DK. Kinect DK can make full use of its depth sensing and RGB image capture capabilities. Especially when dealing with transparent objects, Kinect DK’s depth sensor can help reduce visual interference caused by transparency and reflection, thereby improving the accuracy of object recognition. For the convenience of testing, let *n* = 40. The temporal collection of action photos is represented as $\text{Time}=\left\{t_{1},t_{2},t_{3},\cdots \cdots ,t_{40}\right\}$. Get the brightness, transparency, and color saturation of transparent objects for each action photo. Represents $\text{Lum}=\left\{l_{1},l_{2},l_{3},\cdots \cdots ,l_{40}\right\},\text{Par}=\left\{p_{1},p_{2},p_{3},\cdots \cdots ,p_{40}\right\},\text{Col}=\left\{c_{1},c_{2},c_{3},\cdots \cdots ,c_{40}\right\}$ respectively. Time is expressed as $\text{Time}=\left\{t_{1},t_{2},t_{3},\cdots \cdots ,t_{40}\right\}\text{.}$ Remove missing values from three datasets.

(Equation 15) $$\left\{ \begin{array}{c} {\text{Dt}}_{d1}=\text{Lum}.\text{dropna}\left(\right)\\ {\text{Dt}}_{d2}=\text{Par}.\text{dropna}\left(\right)\\ {\text{Dt}}_{d3}=\text{Col}.\text{dropna}\left(\right) \end{array}\right.$$

where dropna() is a function in the Pandas library. Its role is to remove missing values from the data. ${\text{Dt}}_{d1},{\text{Dt}}_{d2}$ and ${\text{Dt}}_{d3}$ are the datasets after deleting missing values, respectively.

After removing missing values from rough data to prevent clustering, the deleted areas are filled with data so that the data still maintains the original number of samples.

(Equation 16) $$\left\{ \begin{array}{c} {\text{Dt}}_{d1}={\text{Dt}}_{d1}.\text{fillna}\left(\right)\\ {\text{Dt}}_{d2}={\text{Dt}}_{d2}.\text{fillna}\left(\right)\\ {\text{Dt}}_{d3}={\text{Dt}}_{d3}.\text{fillna}\left(\right) \end{array}\right.$$

where fillna() is a function in the Pandas library, which is used to fill in missing values in the data. Data filling of ${\text{Dt}}_{d1},{\text{Dt}}_{d2}$ and ${\text{Dt}}_{d3}$ realizes data update. The sample data is normalized after cleaning.

(Equation 17) $$\left\{ \begin{array}{c} {\text{Dt}}_{s1}=\frac{{\text{Dt}}_{d1}-\min\left({\text{Dt}}_{d1}\right)}{\max\left({\text{Dt}}_{d1}\right)-\min\left({\text{Dt}}_{d1}\right)}\\ {\text{Dt}}_{s2}=\frac{{\text{Dt}}_{d2}-\min\left({\text{Dt}}_{d2}\right)}{\max\left({\text{Dt}}_{d2}\right)-\min\left({\text{Dt}}_{d2}\right)}\\ {\text{Dt}}_{s3}=\frac{{\text{Dt}}_{d2}-\min\left({\text{Dt}}_{d2}\right)}{\max\left({\text{Dt}}_{d3}\right)-\min\left({\text{Dt}}_{d3}\right)} \end{array}\right.$$

where min() and max() are the minimum and maximum samples in the dataset respectively, and ${\text{Dt}}_{s1},{\text{Dt}}_{s2}$ and ${\text{Dt}}_{s3}$ are the normalized datasets respectively. Data are compressed into the range [0, 1].

Analytical hierarchy process (AHP)^26^ is a quantitative analysis method for multi-criteria decision-making problems. In this article, it hierarchizes the preprocessed data of transparent objects and determines the relative weight of each factor by constructing a comparison matrix. You can use the weight distribution formula:

(Equation 18) $$W_{A}\cdot A+W_{B}\cdot B+W_{C}\cdot C$$

where A, B and C represent any sample among ${\text{Dt}}_{s1},{\text{Dt}}_{s2}$ and ${Dt}_{s3}$. $W_{A},W_{B}$ and $W_{C}$ are the weights of A, B, and C, respectively, which satisfy $W_{A}+W_{B}+W_{C}=1$. The purpose is to ensure the standardization of weights. Construct a comparison matrix ($P_{\text{ij}}$).

(Equation 19) $$P_{\text{ij}}=\left[\;\begin{array}{ccc} 1 & \frac{A}{B} & \frac{A}{C}\\ \frac{B}{A} & 1 & \frac{B}{C}\\ \frac{C}{A} & \frac{C}{B} & 1 \end{array}\;\right]$$

Perform eigenvalue decomposition on the comparison matrix and obtain the weight vector as

(Equation 20) $$P_{\text{ij}}\cdot \alpha =\lambda \cdot \alpha$$

where α is the eigenvector corresponding to each eigenvalue. Standardize α to obtain the final weight vector (β). The process of normalization is to divide each component of the feature vector by the sum of all its components to ensure $W_{A}+W_{B}+W_{C}=1$.

(Equation 21) $$\;\text{Result}=\beta_{A}\cdot A+\beta_{B}\cdot B+\beta_{C}\cdot C$$

where Result is the final output result. $\beta_{A},\beta_{B}$, and $\beta_{C}$ are the final weight vectors.

**Remark 2:** This article uses AHP to allocate weights to achieve hierarchical processing. Not only can it give a reasonable relative weight, but it can also reflect the actual influence of each factor in decision-making analysis. It effectively avoids complex information similar to TRANS-AFF.^20^

Perform weighted averaging on chromaticity information.

(Equation 22) $$\text{Avg}=\frac{\beta_{A}\cdot A+\beta_{B}\cdot B+\beta_{C}\cdot C}{\beta_{A}+\beta_{B}+\beta_{C}}$$

**Figure 4:**
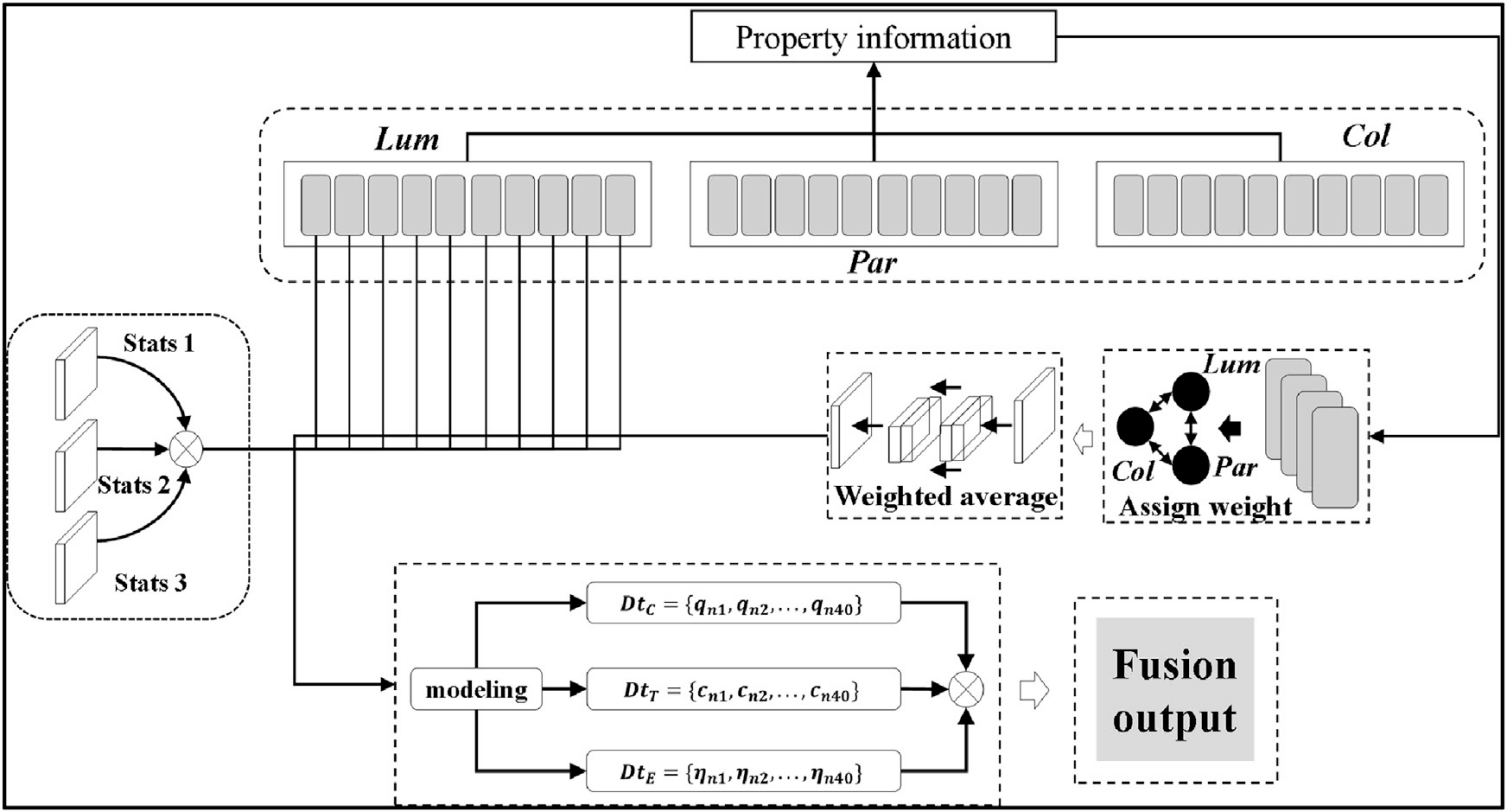
Multimodal dataset design architecture

Construct a multi-modal dataset as shown in Figure 4 to complete image training. The new multimodal dataset is denoted ${\text{Dt}}_{C}=\left\{\;q_{n1},q_{n2},q_{n3},\cdots ,q_{n40}\right\},{\text{Dt}}_{T}=\left\{\;c_{n1},c_{n2},c_{n3},\cdots ,c_{n40}\right\},{\text{Dt}}_{E}=\left\{\eta_{n1},\eta_{n2},\eta_{n3},\cdots ,\eta_{n40}\right\}$.

The multi-modal datasets are fused and a feature image with a size of 100 × 100 pixels is output. A feature matrix is built to represent different samples and features respectively. Map the values in the matrix to pixel values to generate an image. Use the image processing library Matplotlib to create images that represent the values in the matrix as colors. The obtained feature images are introduced into the R-CNN model to extract color change information of transparent objects. When training the R-CNN model, choosing the right hyperparameters has a crucial impact on the performance of the model. The key training hyperparameters used in this article include the following.

The learning rate is set to 0.001, and an adaptive learning rate adjustment algorithm is used. This allows the learning rate to be adjusted dynamically during training, ensuring that the model learns quickly in the early stages and adjusts finely in the later stages. The batch size is set to 32. The number of training rounds is set to 100, and the performance of the validation set determines the optimal number of training rounds to prevent overfitting or underfitting. The momentum is set to 0.9. By introducing the momentum term, we are able to reach the optimal solution faster during training. The weight decay is 0.0005. It helps control the model complexity and ensures that the model does not remember the noise in the training set during training. The cross entropy loss is used as the loss function for the classification task. The formula is as follows:

(Equation 23) $$L\left(y,\hat{y}\right)=-\sum_{i=1}^{n}y_{i}\;\log\left({\hat{y}}_{i}\right)$$

where y is the true label and $\hat{y}$ is the predicted probability.

The regression loss is used to adjust the regression of the bounding box, and the calculation formula is:

(Equation 24) $$L_{\text{reg}}\left(x,y\right)=\sum_{i=1}^{n}{\text{smoot}h}_{L1}\left(x_{i}-y_{i}\right)$$

where ${\text{smoot}h}_{L1}$ is a loss function that linearizes larger errors and squares smaller errors.

The feature image at this time is the feature output of each attribute information. The selective search algorithm can obtain candidate areas of feature images. The target candidate area is set to 500, and the specific process is as follows.

1) Initialize the set of candidate regions and equate the image to the initial candidate region. Similarity calculation is performed by color similarity measure and region size similarity measure.

Select the area with the lowest similarity for segmentation to generate a candidate area. Repeat this step until the target candidate area is 500 and stop the operation. The candidate area information is represented as $\text{region}=\left\{r_{1},r_{2},\cdots \cdots ,r_{500}\right\}$.

The color similarity measure and the region size similarity measure are expressed as follows.

(Equation 25) $$S_{\text{color}}\left(k_{i},k_{j}\right)=\sum_{m=r_{1}}^{r_{500}}\min\left(H_{i}\left(m\right),H_{j}\left(m\right)\right)$$

(Equation 26) $$S_{\text{size}}\left(k_{i},k_{j}\right)=1-\frac{|\text{size}\left(k_{i}\right)-\text{size}\left(k_{j}\right)|}{\max\left(\text{size}\left(k_{i}\right),\text{size}\left(k_{j}\right)\right)}$$

where $k_{i}$ and $k_{j}$ are different neighborhoods of any candidate area. $H_{i}\left(m\right)$ and $H_{j}\left(m\right)$ are the comparison rates of ${\;k}_{i}$ and $k_{j}$ in the m-th candidate area. $\text{size}\left(k_{i}\right)$ and $\text{size}\left(k_{j}\right)$ are the neighborhood areas of $k_{i}$ and $k_{j}$.

Candidate region compression uses fixed ratio compression. Set up 50 classifiers, 500-dimensional features, and 3078 training layers.

(Equation 27) $$\left[\;\begin{array}{ccc} x_{1}^{1} & \;\begin{array}{cc} {\;x}_{2}^{1} & \cdots \end{array} & x_{3078}^{1}\\ x_{1}^{2} & \begin{array}{cc} {\;x}_{2}^{2} & \cdots \end{array} & x_{3078}^{2}\;\\ \begin{array}{c} \cdots \\ x_{1}^{500} \end{array} & \;\begin{array}{cc} \;\begin{array}{c} \cdots \\ x_{2}^{500} \end{array} & \begin{array}{c} \cdots \\ \cdots \end{array} \end{array}\; & \begin{array}{c} \cdots \\ x_{3078}^{500} \end{array} \end{array}\;\right]\;\left[\;\begin{array}{ccc} \omega_{1}^{1} & \cdots & \omega_{1}^{50}\\ \omega_{2}^{1} & \cdots & \omega_{2}^{50}\\ \begin{array}{c} \cdots \\ \omega_{3078}^{1} \end{array} & \begin{array}{c} \cdots \\ \cdots \end{array} & \begin{array}{c} \cdots \\ \omega_{3078}^{50} \end{array} \end{array}\;\right]$$

**Figure 5:**
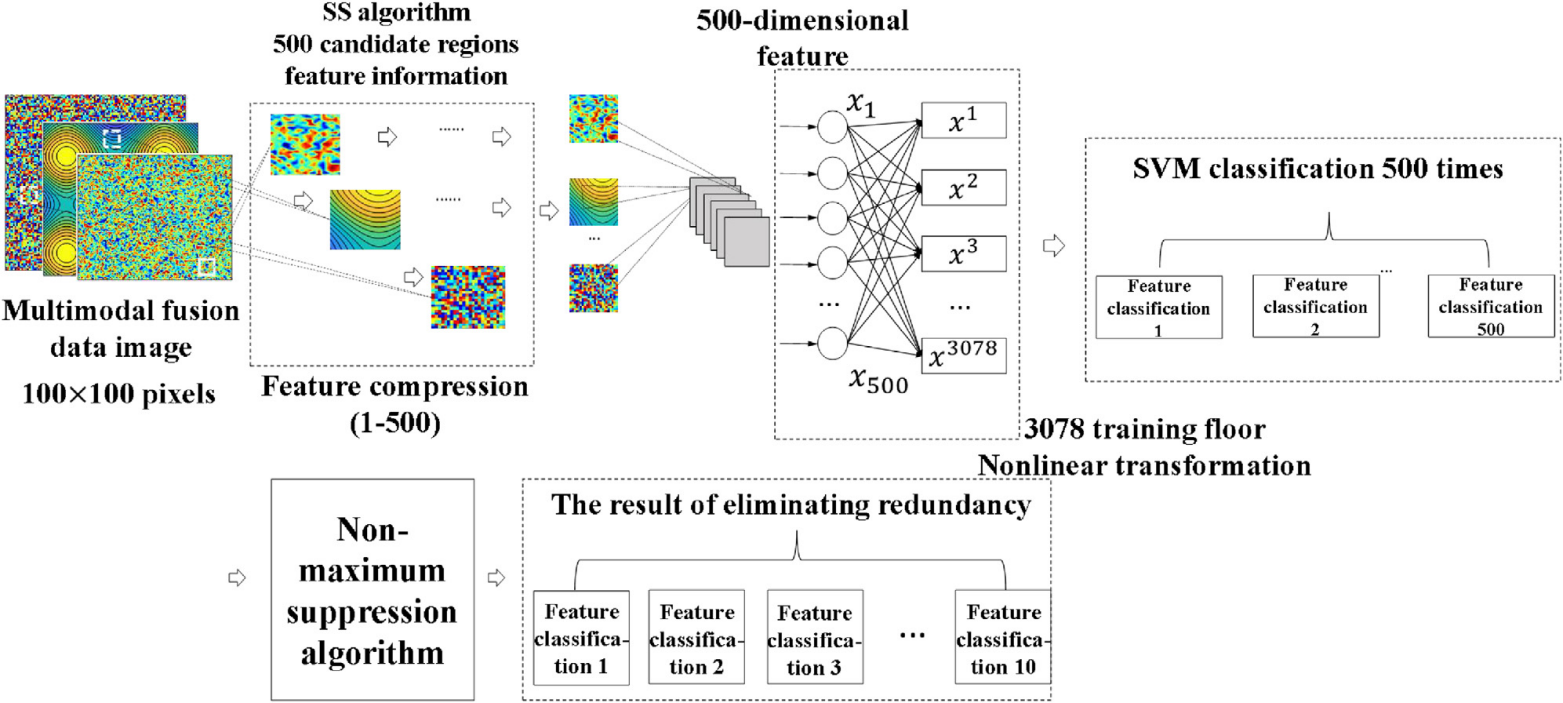
Multi-modal fusion image model

The SVM classification criterion is adopted in the multi-modal fusion image model training in Figure 5. And use the non-maximum suppression algorithm (Non-maximum Suppression) to eliminate repeated features after SVM classification. The candidate area generated by the selective search algorithm is the area with the smallest similarity among the three performance indicators. That is, the candidate area where the color change is most likely to occur. Select 10 feature samples after training in the candidate area, and perform similar superposition, close comparison, and similarity value analysis on them, as shown in Table 1.

**Table 1:** Candidate feature data processing

Data analysis	Different moments nodes									
Luminance	0.51	0.78	0.88	0.83	0.97	0.98	0.88	0.69	0.41	0.2
Transparency	0.24	0.27	0.38	0.42	0.5	0.59	0.68	0.79	0.88	1
Saturation	0.39	0.58	0.63	0.67	0.78	0.89	0.93	0.98	0.99	1
Superposition of similarities	0.24	0.4	0.5	0.32	0.38	0.18	0.1	0.58	1.16	1.6
Draw a close comparison	0.42	0.43	0.39	0.96	1.11	1.92	2.19	0.72	1.2	2.6
Similarity Analysis 1	0.599		0.0354		0.1878		0.0366		0.0976	
Similarity Analysis 2		0.319		0.4746		0.0366		0.2202		

**Remark 3:** The key to this step is to use a similar feature extraction method to monitor color changes by detecting whether the properties of transparent objects deviate from the normal range. This idea makes monitoring more flexible and effective and provides a new method for capturing transparent objects, thereby realizing the judgment of color change information of transparent objects.

### Adaptive imitation synthesis (AISP)

The three key time nodes for secondary grabbing are set as $t_{s},t_{m}$ and $t_{e}$. They respectively represent the grabbing start time, the rotation occurrence time and the grabbing end time. The dynamic trajectory equation of the entire process can be expressed as,

(Equation 28) $$P\left(t\right)=f\left(\;{t,t}_{s},t_{m},t_{e}\right)$$

where P(t) is the real-time location of the grabbing device. f is the motion characteristic function. According to (1) and (2), the dynamic trajectory of the secondary grabbing node set is established.

(Equation 29) $$G_{cup}\left(t\right)=\left\{ \begin{array}{cc} \left(x_{\text{start}1},y_{\text{start}1},z_{\text{start}1}+v_{\text{up}1}\left(t-t_{s}\right)\right), & t_{s}<t<t_{m}\\ \left(x_{\text{rotatel}1}\left(t-t_{m}\right),y_{\text{rotatel}1}\left(t-t_{m}\right),z_{\text{rotatel}1}\left(t-t_{m}\right)\right), & t_{m}<t<t_{e}\\ \left(x_{\text{down}1},y_{\text{down}1},z_{\text{down}1}-v_{\text{down}1}\left(t-t_{e}\right)\right), & t>t_{e} \end{array}\right.$$

where $v_{\text{up}1}$ and $v_{\text{down}1}$ represent the rising and falling speeds.

The dynamic trajectory tracking equations of $P_{\text{cup}}$ and $I_{\text{cup}}$ satisfy,

(Equation 30) $${\;P}_{cup}\left(t\right)/I_{cup}=\left\{ \begin{array}{cc} \left(x_{\text{start}2}+f\left(t\right),y_{\text{start}2},z_{\text{start}2}\right), & t_{s}<t<t_{m}\\ \left(x_{\text{rotatel}2}+f\left(t_{m}\right)\cos\left(g\left(t\right)\right),y_{\text{rotatel}2}+f\left(t_{m}\right)\sin\left(g\left(t\right)\right),z_{\text{rotatel}2}\right), & t_{m}<t<t_{e}\\ \left(x_{\text{down}2}+f\left(t_{m}\right)\cos\left(f\left(t_{e}\right)\right)+h\left(t\right),y_{\text{down}2}+f\left(t_{m}\right)\sin\left(f\left(t_{e}\right)\right),z_{\text{down}2}\right), & t>t_{e} \end{array}\right.$$

g(t) is the rotation function. h(t) is the excitation function. $f\left(t_{m}\right)$ and $f\left(t_{e}\right)$ are the rising trajectory function and falling trajectory function at ${\;t}_{m}$ and ${\;t}_{e}$ moments respectively.

Establish a mapping relationship between dynamic trajectory tracking equations and object attribute information in time series to form a comprehensive dataset (Data).

(Equation 31) $$\text{Data}=\left\{ \begin{array}{cc} G_{\text{cup}}\left(t_{1}\right)=\left(x_{1},y_{1},z_{1},{\dot{x}}_{1},{\dot{y}}_{1},{\dot{z}}_{1}\right), & t=t_{1}\\ ......, & \;......\\ G_{\text{cup}}\left(t_{40}\right)=\left(x_{40},y_{40},z_{40},{\dot{x}}_{40},{\dot{y}}_{40},{\dot{z}}_{40}\right), & t=t_{40}\\ P_{\text{cup}}\left(t_{1}\right)=\left(x_{1},y_{1},z_{1},{\dot{x}}_{1},{\dot{y}}_{1},{\dot{z}}_{1}\right), & t=t_{1}\\ \;......, & ......\\ P_{\text{cup}}\left(t_{40}\right)=\left(x_{40},y_{40},z_{40},{\dot{x}}_{40},{\dot{y}}_{40},{\dot{z}}_{40}\right), & t=t_{40}\\ \left(\text{Lum}\left(t_{1}\right),\text{Par}\left(t_{1}\right),\text{Col}\left(t_{1}\right)\right), & t=t_{1}\\ ......, & ......\\ \left(\text{Lum}\left(t_{40}\right),\text{Par}\left(t_{40}\right),\text{Col}\left(t_{40}\right)\right), & t=t_{40} \end{array}\right.$$

where $x_{i},y_{i\;},z_{i},\dot{x_{i}},\dot{y_{i},}\dot{z_{i}}$ are the trajectory position and speed at time t = i respectively.

**Figure 6:**
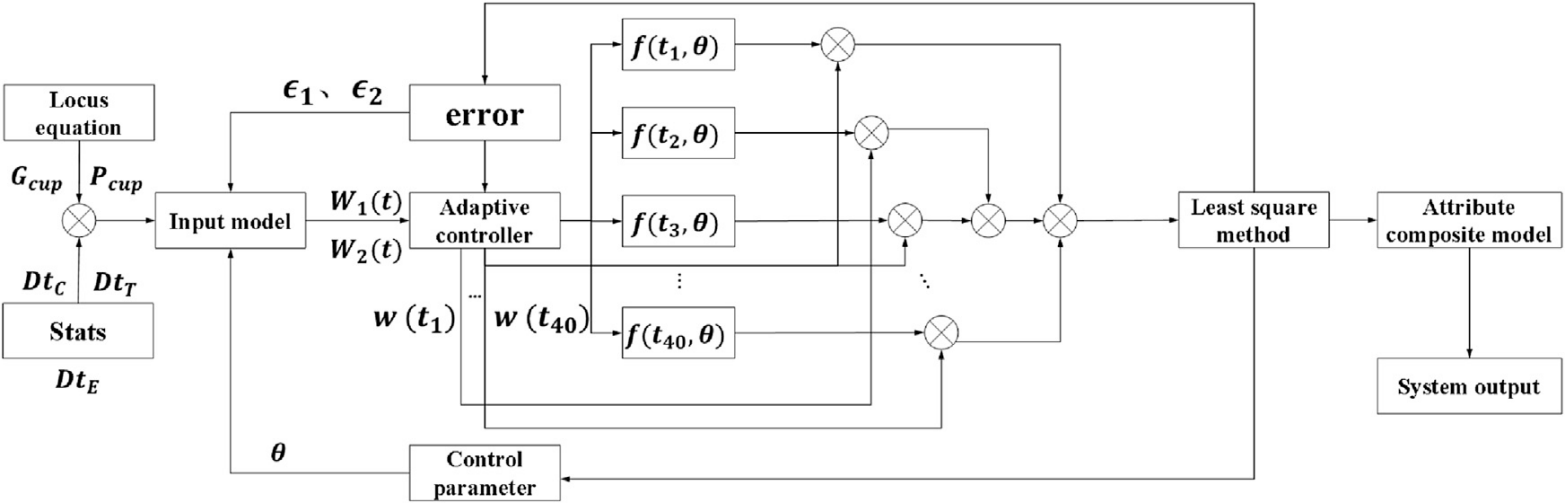
Adaptive control system

Design an adaptive control system, as shown in Figure 6. The system is trained using the established comprehensive dataset. The control parameters are determined by learning the information association between dynamic trajectory information and object attributes. Set up the system debugging function during the capture process and establish a system debugging model in the form of the system debugging function.

(Equation 32) $$W_{1}\left(t\right)=G_{\text{cup}}\left(t\right)+f\left(t,\theta \right)+\epsilon_{1}$$

(Equation 33) $$W_{2}\left(t\right)=P_{\text{cup}}\left(t\right)+f\left(t,\theta \right)+\epsilon_{2}$$

where $W_{1}\left(t\right)$ is the system debugging function of the grab point. $W_{2}\left(t\right)$ is the system debugging function of the central point and internal key nodes. f(t,θ) is the dynamic model of the control parameter θ at time t. Both $\epsilon_{1}$ and $\epsilon_{2}$ are model errors. Trajectory tracking equations and attribute information serve as inputs to the adaptive control system.

According to the dynamic trajectory dataset, $\theta {,\epsilon }_{1}$ and $\epsilon_{2}$ are determined by the least squares method. $\theta {,\epsilon }_{1}$ and $\epsilon_{2}$ real-time feedback input model. Establish the time correspondence between system debugging functions: $W_{t1}=\{\left(t_{1},W_{1}\left(t_{1}\right)\right),\left(t_{2},W_{1}\left(t_{2}\right)\right),\left(t_{3},W_{1}\left(t_{3}\right)\right),\cdots ,\left(t_{40},W_{1}\left(t_{40}\right)\right)\}$。 $W_{t2}=\left\{\left(t_{1},W_{2}\left(t_{1}\right)\right),\left(t_{2},{\;W}_{2}\left(t_{2}\right)\right),\left(t_{3},{\;W}_{2}\left(t_{3}\right)\right),\cdots ,\left(t_{40},W_{2}\;\left(t_{40}\right)\right)\right\}.\;W\left(t\right)=\left\{\left(t_{1},W\left(t_{1}\right)\right),\left(t_{2},W\left(t_{2}\right)\right),\left(t_{3},W\left(t_{3}\right)\right),\cdots ,\left(t_{40},W\left(t_{40}\right)\right)\right\}$.

A loss function is introduced to minimize the objective function through the sum of squares of the residuals.

(Equation 34) $$\left\{ \begin{array}{c} J\left(\theta ,\epsilon_{1}\right)=\sum_{i=1}^{40}{\left[W_{1}\left(t\right)-f\left(t_{i},\theta \right)-\epsilon_{1}\right]}^{2}\\ J\left(\theta ,\epsilon_{2}\right)=\sum_{i=1}^{40}{\left[W_{2}\left(t\right)-f\left(t_{i},\theta \right)-\epsilon_{2}\right]}^{2} \end{array}\right.$$

Let the partial derivative of the minimized objective function be equal to zero.

(Equation 35) $$\left\{ \begin{array}{c} \partial \left(\;J\left(\theta ,\epsilon_{1}\right)\right)/\partial \theta =0\\ \partial \left(\;J\left(\theta ,\epsilon_{1}\right)\right)/\partial \epsilon_{1}=0\\ \partial \left(\;J\left(\theta ,\epsilon_{2}\right)\right)/\partial \theta =0\\ \partial \left(\;J\left(\theta ,\epsilon_{2}\right)\right)/\partial \epsilon_{2}=0 \end{array}\right.$$

The normal equation of the least squares method can be obtained through (8):

(Equation 36) $$Y^{T}Y\left[\;\begin{array}{c} \theta \\ \epsilon_{1}\\ \epsilon_{2} \end{array}\;\right]=Y^{T}S$$

where the size of Y is a 3 × 3 design matrix. It contains the relevant values of θ. S is the information vector of object attributes of ${\text{Dt}}_{C}\left(t_{i}\right),{\text{Dt}}_{T}\left(t_{i}\right)$ and ${\text{Dt}}_{E}\left(t_{i}\right)$ at $t_{i}$.

Based on the above information, the parameters are continuously adjusted with the help of the least squares method. $\theta {,\epsilon }_{1}$ and $\epsilon_{2}$ are finally determined. Establish a transparent object attribute model based on the determined parameters.

(Equation 37) $$\left\{ \begin{array}{c} B\left(t\right)=q_{k}\cdot e^{-\alpha t}\\ T\left(t\right)=c_{k}\cdot e^{-\beta t}\\ S\left(t\right)=\eta_{k}\cdot e^{-\gamma t}\; \end{array}\right.$$

where $q_{k}$ is any brightness sample in ${\text{Dt}}_{C}=\left\{q_{n1},q_{n2},q_{n3},\cdots \cdots ,q_{n40}\right\}\text{.}$ α is the attenuation coefficient related to optical properties and ambient lighting. B(t) represents the time change function of brightness. $c_{k}$ is any transparency sample in ${\text{Dt}}_{T}=\left\{c_{n1},c_{n2},c_{n3},\cdots \cdots ,c_{n40}\right\}\text{.}$ β is the transparency attenuation coefficient. T(t) represents the time change function of transparency. $\eta_{k}$ is any color saturation sample in ${\text{Dt}}_{E}=\left\{\eta_{n1},\eta_{n2},\eta_{n3},\cdots \cdots ,\eta_{n40}\right\}\text{.}$ γ is the color saturation attenuation coefficient. S(t) represents the time change function of color saturation. The trajectory and attribute composite model of a transparent object are as follows.

(Equation 38) $$\left\{ \begin{array}{c} F_{1}\left(B,T,S,\lambda \right)=B\left(t\right)W_{1}\left(t\right)\cdot T\left(t\right)W_{1}\left(t\right)\cdot S\left(t\right)W_{1}\left(t\right)+W_{2}\left(t\right)\cdot \lambda \\ F_{2}\left(B,T,S,\lambda \right)=B\left(t\right)W_{2}\left(t\right)\cdot T\left(t\right)W_{2}\left(t\right)\cdot S\left(t\right)W_{2}\left(t\right)+W_{1}\left(t\right)\cdot \lambda \\ \lambda =\frac{\alpha }{\beta }+\frac{\beta }{\gamma }+\frac{\gamma }{\alpha } \end{array}\right.$$

where $F_{1}\left(B,T,S,\lambda \right)$ and ${\;F}_{2}\left(B,T,S,\lambda \right)$ are composite functions about the trajectory and attribute model of transparent objects. λ is a constant.

Based on the above information, the adaptive control system state vector is established.

(Equation 39) $$\text{Vec}\left(t\right)=\left[F_{1}\left(B,T,S,\lambda \right),{\;F}_{2}\left(B,T,S,\lambda \right)\right]$$

We design an adaptive controller.

(Equation 40) $$\nu \left(t\right)={Κ}_{p}\cdot e\left(\epsilon_{1},\epsilon_{2}\right)+{Κ}_{d}\cdot \text{Vec}\left(t\right)+{Κ}_{i}\cdot \int e\left(\epsilon_{1},\epsilon_{2}\right)\text{dt}$$

where ν(t) is the controller output. $e\left(\epsilon_{1},\epsilon_{2}\right)$ is the dynamic trajectory error. ${Κ}_{p}$ is the proportional gain. It determines the proportional relationship between ν(t) and $e\left(\epsilon_{1},\epsilon_{2}\right)$. ${Κ}_{d}$ is the differential gain. It determines the response relationship between ν(t) and Vec(t). ${Κ}_{i}$ is the integral gain. It reflects the integral relationship between ν(t) and $e\left(\epsilon_{1},\epsilon_{2}\right)$ and has the function of eliminating steady-state errors.

According to (15), ${\;Κ}_{i}\cdot \int e\left(\epsilon_{1},\epsilon_{2}\right)\text{dt}$ is expanded and the saturation function O(x) is introduced. It is used to control the change growth of ${\;Κ}_{i}\cdot \int e\left(\epsilon_{1},\epsilon_{2}\right)\text{dt}\;\text{.}$

(Equation 41) $$\nu \left(t\right)={Κ}_{p}\cdot e\left(\epsilon_{1},\epsilon_{2}\right)+{Κ}_{d}\cdot \text{Vec}\left(t\right)+\int O\left({Κ}_{i}\cdot \int e\left(\epsilon_{1},\epsilon_{2}\right)\text{dt}\right)\text{dt}$$

Adaptive gains ${Κ}_{\alpha },{Κ}_{\beta }$ and ${Κ}_{\gamma }$ are introduced in the adaptive adjustment mechanism. It can complete the online state adjustment in transparent object grasping. The adaptive gain relationship of proportional gain, differential gain and integral gain changing with time is established.

(Equation 42) $$\left\{ \begin{array}{c} {Κ}_{p}\left(t\right)={Κ}_{\alpha }\cdot {Κ}_{p0}\\ {Κ}_{d}\left(t\right)={Κ}_{\beta }\cdot {Κ}_{d0}\\ {Κ}_{i}\left(t\right)={Κ}_{\gamma }\cdot {Κ}_{i0} \end{array}\right.$$

where ${Κ}_{p0},{Κ}_{d0}$ and ${Κ}_{i0}$ are the gain coefficients before the time change. Based on the above information, the design process of the adaptive control system is shown in Table 2.

**Table 2:** Adaptive control system design process

**Iput**: $G_{cup}\left(t\right),P_{cup}\left(t\right),{Dt}_{C},{Dt}_{T},{Dt}_{E},Vec\left(t\right),v\left(t\right),W\left(t\right),e\left(\epsilon_{1},\epsilon_{2}\right)$ Learning rate(learning_rate), Number of iterations(I), Maximum Iterations(MI), Allowance(TE), Control parameyer(θ)
Step1: $G_{cup}\left(t\right)=\text{compute}\_\text{trajectory}\left(\text{Para}\right)$ Step2: $P_{cup}\left(t\right)=\text{compute}\_\text{trajectory}\_\text{properties}\left(G_{cup}\left(t\right)\right)$ Step3: ${Dt}_{C},{Dt}_{T},{Dt}_{E}=\text{compute}\_\text{attributes}\left(P_{cup}\left(t\right)\right)$ Step4: $I=1:e\left(\epsilon_{1},\epsilon_{2}\right)=e\left(\epsilon_{1},\epsilon_{2}\right)\_\text{function}\left(G_{cup}\left(t\right),P_{cup}\left(t\right)\right)$ Step5: Calculated gradient: Grad = Gradient($e\left(\epsilon_{1},\epsilon_{2}\right)$, W(t)) Step6: Update parameter: θ=θ−learning_rate∗grad Step7: Calculate the new dynamic trajectory error: $\text{new}\_e\left(\epsilon_{1},\epsilon_{2}\right)=e\left(\epsilon_{1},\epsilon_{2}\right)\_\text{function}\left(\theta \right)$ Step8: if($|\text{new}\_e\left(\epsilon_{1},\epsilon_{2}\right)|<\text{TE}$) I=I+1 $Vec\left(t\right)=\left[G_{cup}\left(t\right),P_{cup}\left(t\right),{Dt}_{C},{Dt}_{T},{Dt}_{E}\right]$ v(t)=ompute_contro_output(optimized_θ,Vec(t)) Step9: if(I>MI) Outputoptimalcontrolparameters→θ Outoftheloop

**Figure 7:**
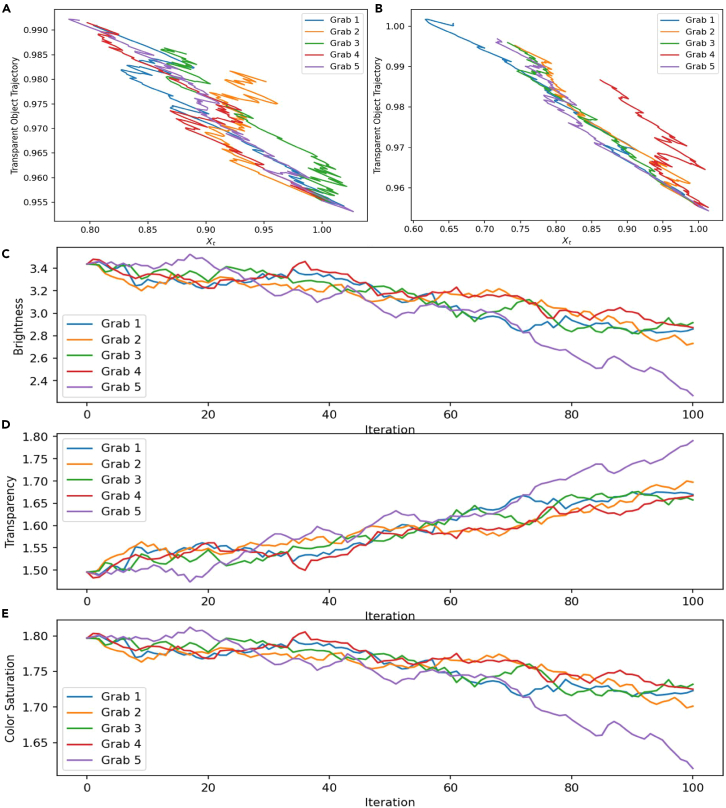
Iteration of transparent object trajectory and attribute information (A and B) is the trajectory iteration of 5 grabs. (C–E) is the attribute iteration of 5 grabs.

In the adjustment parameters of the adaptive control system, the iterative process of grabbing trajectories and attribute information for different transparent objects is shown in Figure 7.

Here, a total of five grabbing processes from Grab1 to Grab5 are simulated. During the iteration process of the transparent object trajectory, the horizontal axis represents the discrete steps of time. The total simulation time is 1 min, divided into 1000 steps. Trajectory iterations gradually converge to a fit with discrete steps. Reflects the process of optimizing parameter adjustment. The error of object attribute information in 100 iterations is within a small range.

It can be seen from Equations 2 and 3 that $G_{\text{cup}},{\;P}_{\text{cup}}$ and $I_{\text{cup}}$ are three-dimensional trajectories parameterized with respect to time. That is $G_{\text{cup}}\left(t\right)=\left[x\left(t\right),y\left(t\right),z\left(t\right)\right],{\;P}_{\text{cup}}\left(t\right)=\left[x\left(t\right),y\left(t\right),z\left(t\right)\right]{,I}_{\text{cup}}\left(t\right)=\left[x\left(t\right),y\left(t\right),z\left(t\right)\right]\text{.}$ Establish a relationship with θ. That is ${\;G}_{\text{cup}}\left(t,\theta \right){,P}_{\text{cup}}\left(t,\theta \right),I_{\text{cup}}\left(t,\theta \right)\text{.}$ Establish a relationship between the three.

(Equation 43) $$\begin{array}{l} G_{cup}\left(t,\theta \right)={\mathrm{argmin}}_{\theta }\sum_{para=1}^{MI}\|{\;P}_{cup}\left({Dt}_{C}\left[para\right],\theta \right),I_{cup}\left(\;{Dt}_{T}\left[para\right],\theta \right),{Dt}_{E}\left(para,\theta \right)\| \end{array}$$

where para is a constant. ${\text{Dt}}_{C}\left[\text{para}\right]=q_{\text{para}-1},{\text{Dt}}_{T}\left[\text{para}\right]=c_{\text{para}-1},{{\text{Dt}}_{E}}_{\text{para}}=\eta_{\text{para}-1}\text{.}$ ${\text{Dt}}_{E}\left(\text{para},\theta \right)$ represents the color saturation of $\eta_{\text{para}-1}$ related to the control parameter.

The changes in attributes over time and control parameters during learning can be continuously adjusted according to (18). The adjustment process is as follows.

(Equation 44) $${\text{Dt}}_{C}\left(t,\theta \right)=\frac{1}{n}\sum_{\text{para}=1}^{40}{\left(\frac{\partial G_{\text{cup}}\left(t,\theta \right)}{\partial t}\right)}^{2}$$

(Equation 45) $${\text{Dt}}_{T}\left(t,\theta )=\exp(-\int_{\text{para}=1}^{40}\frac{\partial G_{\text{cup}}\left(t,\theta \right)}{\partial \theta }\right)$$

(Equation 46) $${\text{Dt}}_{E}\left(t,\theta \right)=\sqrt{\sum_{\text{para}=1}^{40}{\left(\frac{\partial G_{\text{cup}}\left(t,\theta \right)}{\partial t}\right)}^{2}+{\left(\frac{\partial G_{\text{cup}}\left(t,\theta \right)}{\partial \theta }\right)}^{2}}$$

At this time, the system debugging function W(t) can be used to quantify the dynamic trajectory error $e\left(\epsilon_{1},\epsilon_{2}\right)$.

(Equation 47) $$W\left(t,\theta \right)=\sum_{\text{para}=1}^{40}{\left({\;G}_{\text{cup}}\left(t,\theta \right)-e\left(\epsilon_{1},\epsilon_{2}\right)\;\right)}^{2}+{\left({\;P}_{\text{cup}}\left(t\right)-e\left(\epsilon_{1},\epsilon_{2}\right)\right)}^{2}+{\left({\;I}_{\text{cup}}\left(t\right)-e\left(\epsilon_{1},\epsilon_{2}\right)\right)}^{2}$$

Then the state vector Vec(t) in the adaptive control system synthesizes the above data information.

(Equation 48) $$\text{Vec}\left(t\right)=\left[\begin{array}{c} {\;G}_{\text{cup}}\left(t,\theta \right)\\ \begin{array}{c} \;{\text{Dt}}_{C}\left(t,\theta \right)\\ \begin{array}{c} {\text{Dt}}_{T}\left(t,\theta \right)\\ \;{\text{Dt}}_{E}\left(t,\theta \right) \end{array} \end{array} \end{array}\right]$$

**Figure 8:**
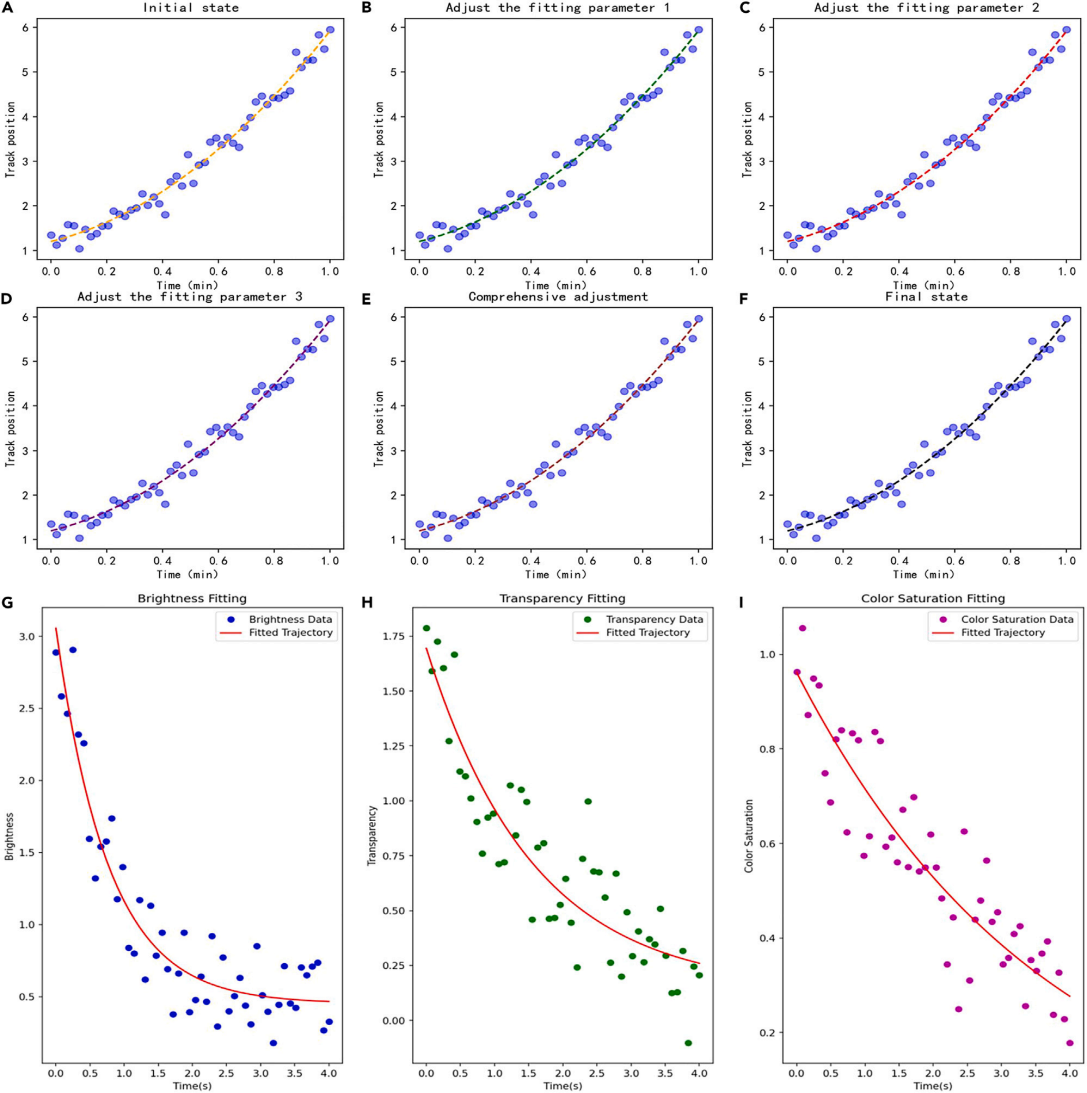
Fitting effect after averaging the trajectory and attribute data (A–F) is the trajectory fitting effect under different parameter states. (G–I) is the attribute fitting effect under different parameter states.

Figure 8 averages multiple capture data of trajectories and attributes through adaptive imitation learning. The fitting effect is shown.

Figures 8A–8F shows that as the parameters are adjusted, the trajectory fitting effect becomes more and more concentrated. This shows that the model can better capture the data characteristics and patterns of the trajectory. The fitting effect in Figures 8G–8I also confirms this.

## Results

The proposed technology is compared with two traditional grasping technologies. They are the lightweight robot grasping technology based on template matching depth image^27^ (TMDI) and the grasping technology based on visual recognition processing^28^ (VRP).

**Figure 9:**
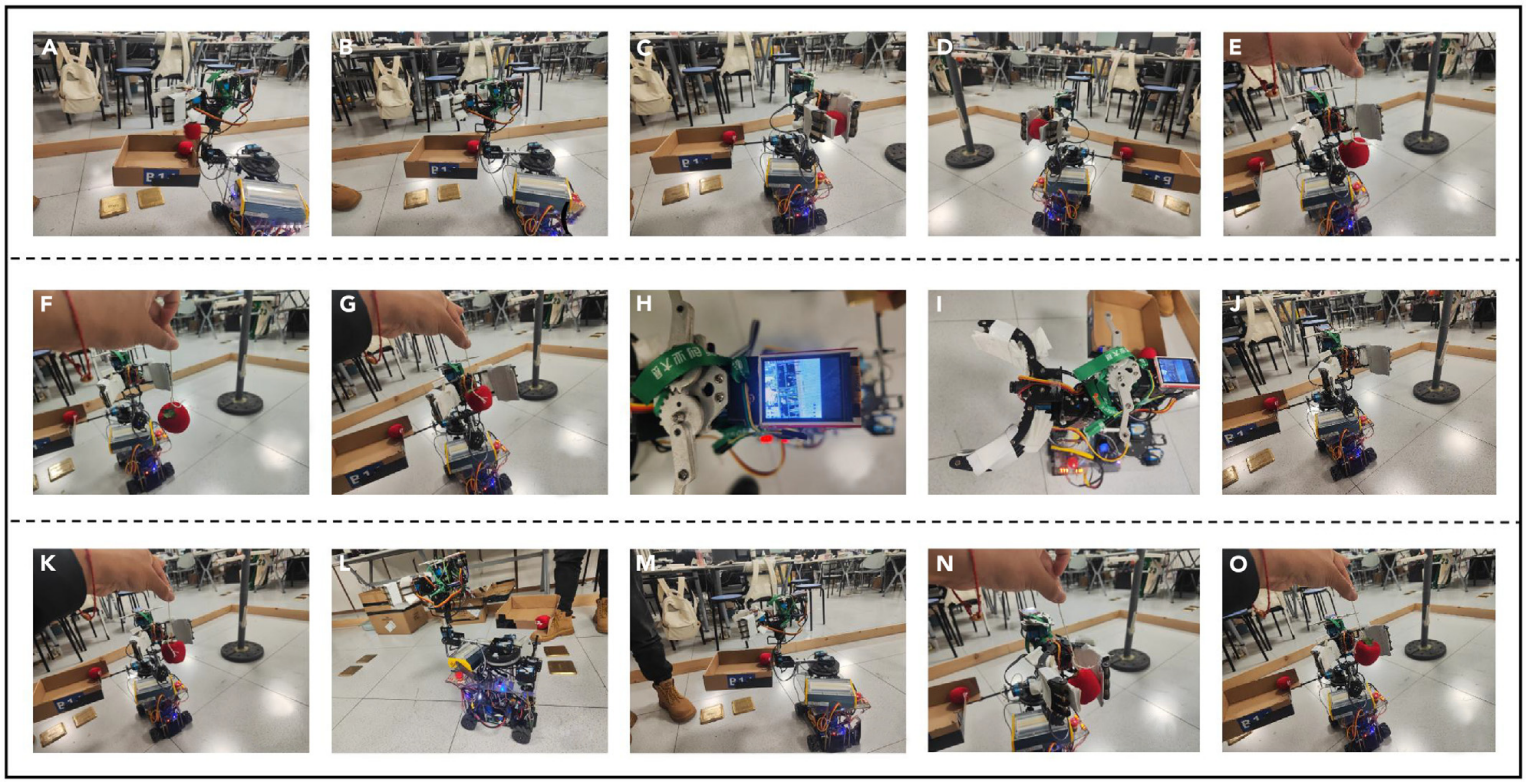
Physical grabbing test (A–E) is the grabbing test at angle 1. (F–J) is the grabbing test at angle 2. (K–O) is the grabbing test at angle 3. The grabbing environment at different angles is different.

Since the two methods cannot be directly compared with the proposed method, we considered the more mainstream generative grasping network for comparison. The grasping comparison test is implemented with the help of this generative grasping network. A transparent glass cup is a typical color-changing object. Its color is different under different lighting or background. Since the front-end design of the grasping robot in the laboratory belongs to the "soft robot," it is more suitable for grasping soft transparent objects. Due to the characteristics of the transparent glass cup itself and the limitations of the laboratory lighting test environment, we use colored balls of the same shape but different colors to replace the transparent glass cup. These balls of different colors can be equivalent to different color changes of the same transparent object. Their color changes are more obvious, which is conducive to experimental testing, as shown in Figure 9.

Set the total number of grasps to 40. Consider five basic scenarios.

1) Scenario 1: only contains the object to be grasped, no interference information, and no color change.
2) Scenario 2: There is interference information, but no color change.
3) Scenario 3: There is interference information and there is color change.
4) Scenario 4: The interference information is more complex and there is no color change.
5) Scenario 5: The interference information is more complex and there is color change.

**Figure 10:**
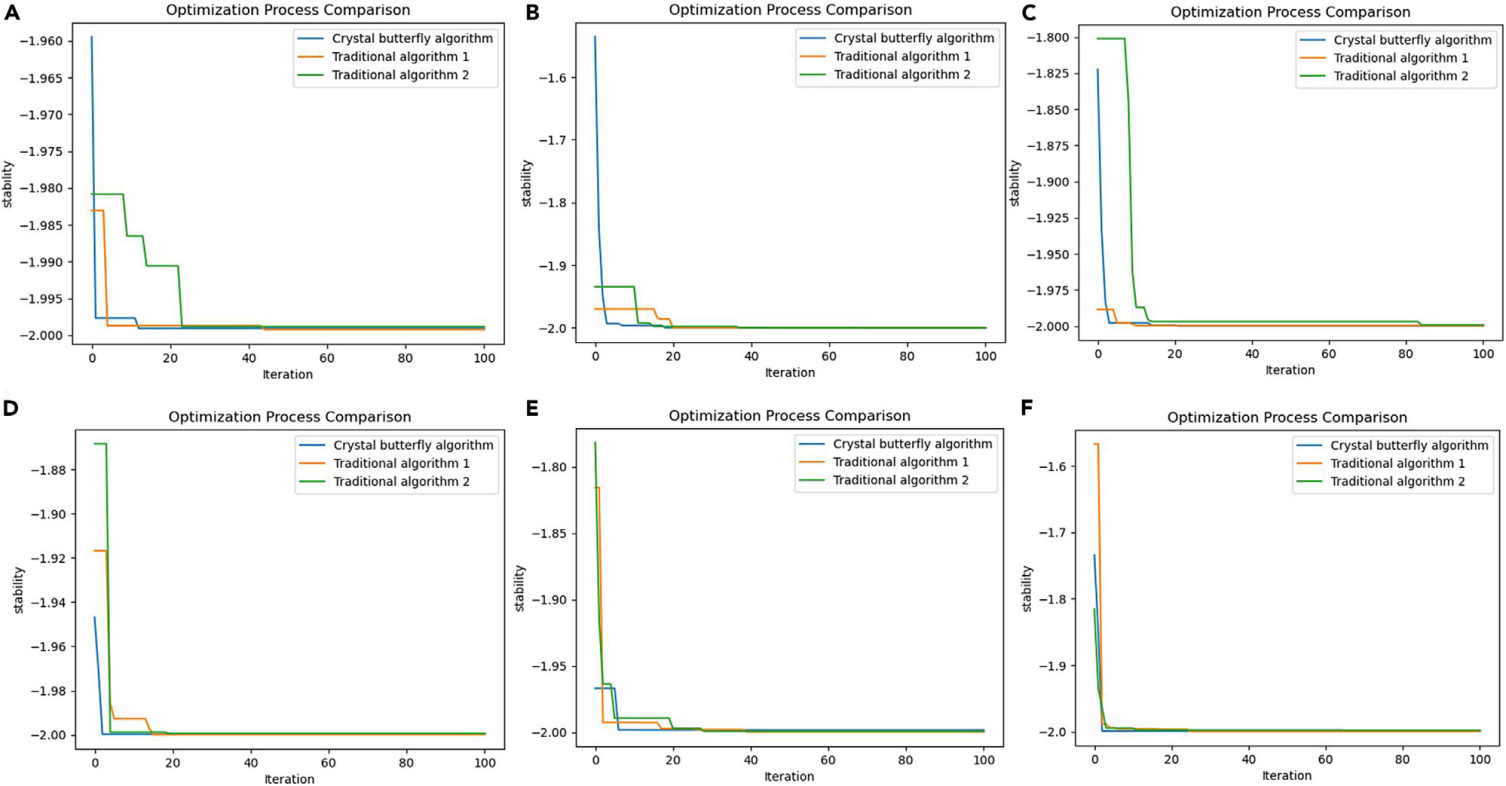
Stability comparison of different algorithms (A–F) are the results of multiple tests on the stability of TMDI, VRP and the algorithm proposed in this article.

Figure 10 shows the test process. We compared the success rate and average cycle of 40 grasping, as shown in Tables 3 and 4. The tabular data shows that in the grasping environment without color change, the grasping success rate of the three methods is relatively high. VPR and the method in this article both reached 92.5%. The grasping success rate of TMID in scenario 2 is higher than that in scenario 1, indicating the instability of the system. Once color changes and complex interference occur, the grasping success rates of TMID and VPR drop significantly. However, the method in this article still maintains a relatively high grasping success rate. Its average grasping success rate is stable at 86.5%. The average grasping cycle is stable at 18.66s.

**Table 3:** Comparison of grasping success rates across 5 scenarios

Scene	The number of grabs *N* = 40				
	Scene 1	Scene 2	Scene 3	Scene 4	Scene 5
TMDI	85%	90%	55%	75%	22.5%
VPR	92.5%	87.5%	40%	82.5%	42.5%
This method	92.5%	90%	85%	87.5%	77.5%

**Table 4:** Comparison of average grasping cycle times across 5 scenarios

Scene	The number of grabs *N* = 40				
	Scene 1	Scene 2	Scene 3	Scene 4	Scene 5
TMDI	17.61s	23.18s	22.68s	24.32s	23.59s
VPR	21.47s	20.57s	24.29s	24.70s	27.46s
This method	10.03s	16.92s	19.66s	23.04s	23.65s

We tested and compared the algorithm stability during grabbing. A stable grasping algorithm can produce consistent results in different scenarios and initial conditions. This consistency is crucial for application in practical environments. TMDI and VRP are sensitive to initial conditions and are prone to fall into local optimal solutions in certain scenarios, resulting in the stagnation of grasping accuracy for a period of time. The Crystal Butterfly algorithm proposed in this article is more likely to stabilize during the iteration process, and different test results are shown in Figure 10. The number of iterations of TMDI and VRP is mostly concentrated around 20 times or even more than 20 times. The number of iterations of the method in this article is less than 20 times. Multiple test results also confirm this point.

## Discussion

This article studies a crystal butterfly algorithm and adaptive imitation synthesis recognition and grabbing technology. The proposed butterfly trajectory dynamic node tracking method achieves accurate tracking and real-time adjustment of transparent object trajectories, effectively improving the trajectory detection and tracking effect. Combined with color dynamic recognition technology, it successfully overcomes the recognition bias caused by color changes. And improve the accuracy and efficiency of multi-angle feature extraction. Finally, adaptive imitation synthesis technology is introduced to make up for the shortcomings of traditional imitation learning such as poor data adaptability and simple copying, and realize the promotion of multi-scenario grabbing applications.

### Limitations of the study

The grasping device in this article is a single agent. In practical applications, collaborative applications between multiple agents are often involved. When a transparent body requires the collaborative operation of multiple machines, multi-dimensional and complex nonlinear terms will be involved. For these nonlinear terms, the least squares identification scheme is an interesting test. Designing relevant estimators and controllers based on the least squares identification scheme to achieve the global stability of the nonlinear system is a feasible solution. In the future, we plan to extend this technology to the application scenario of multi-agent collaborative grasping. And design a new least squares identification scheme and apply it to more complex multi-dimensional nonlinear systems to achieve collaborative control.

## STAR★Methods

### Key resources table

**Table:** 

REAGENT or RESOURCE	SOURCE	IDENTIFIER
**Software and algorithms**		

PyCharm Community Edition 2023.2.5	Python Software Foundation	https://www.python.org
MATLAB R2022a	Matlab Software Foundation	https://ww2.mathworks.cn/products/matlab-home.html
Selective Search algorithm	This manuscript	N/A
Butterfly Optimization Algorithm	This manuscript	N/A
Attribute Supply Material 1	https://data.mendeley.com/datasets/x9yw8k77vd/1	https://doi.org/10.17632/x9yw8k77vd.1
Attribute Supply Material 2	https://data.mendeley.com/datasets/x9yw8k77vd/1	https://doi.org/10.17632/x9yw8k77vd.1
Track information supply materials	https://data.mendeley.com/datasets/x9yw8k77vd/1	https://doi.org/10.17632/x9yw8k77vd.1
Test Code	https://zenodo.org/records/12542227	https://doi.org/10.5281/zenodo.12542227

**Other**		

Stacking and unstacking equipment	Anqiu Boyang Machinery Manufacturing Co., Ltd	http://www.sdbyjx.net
Grab device	Robotics R&D Laboratory of Shandong Technology and Business University	N/A
Financial support	Shandong Provincial Technology Innovation Guidance Program	YDZX2023030
Depth camera	Kinect DK	N/A
R-CNN Model	This manuscript	N/A
CDR	This manuscript	N/A
Multi-modal fusion image model	This manuscript	N/A

### Resource availability

#### Lead contact

Further information and requests for resources and reagents should be directed to and will be fulfilled by the lead contact, Chuanyu Cui (cuichuanyu1999@163.com).

#### Materials availability

This study did not generate new unique reagents.

#### Data and code availability

• In the grabbing test of dismantling and stacking, the route data of butterfly trajectory and transparent object attribute data have been deposited at Mendeley Data, which are publicly available as of the date of publication. The DOI is listed in the key resources table.
• All original code has been deposited at Zenodo and is publicly available as of the date of publication. DOIs are listed in the key resources table.
• Any additional information required to reanalyze the data reported in this paper is available from the lead contact upon request.

### Experimental model and study participant details

#### Intelligent grasping experiment

The experimental part of the grasping test in this study was carried out in the Robot Control Laboratory of Shandong Technology and Business University and verified by Anqiu Boyang Machinery Manufacturing Co., Ltd.

Model Design: This study introduces a recognition and grasping technique for color-variable objects based on the Crystal Butterfly Algorithm and Adaptive Imitation Synthesis. The primary experimental subject is a transparent glass, with colored balls used to simulate color changes under different lighting and background conditions. The experimental setup includes a depth camera (Kinect DK) and a soft robotic gripper.

Data Acquisition: Data on the brightness, transparency, and color saturation of transparent objects were collected using the Kinect DK depth camera. Specific experimental parameters are as follows:

Number of Photos: 40.

Time set: $\text{Time}=\left\{t_{1},t_{2},\cdots ,t_{40}\right\}\text{.}$ Brightness Data Set: $\text{Lum}=\left\{l_{1},l_{2},\cdots ,l_{40}\right\}\text{.}$ Transparency Data Set: $\text{Par}=\left\{p_{1},p_{2},\cdots ,p_{40}\right\}\text{.}$ Color Saturation Data Set: $\text{Col}=\left\{c_{1},c_{2},\cdots ,c_{40}\right\}\text{.}$

Study Subjects: The primary subject of the study is a transparent glass, with colored balls simulating color changes under various lighting and background conditions to test the grasping success rate and cycle in different scenarios.

Experimental Scenarios:

Scenario 1: Only the target object is present, no interference, no color change.

Scenario 2: Interference is present, but no color change.

Scenario 3: Interference and color change are present.

Scenario 4: More complex interference, no color change.

Scenario 5: More complex interference and color change.

Data Processing and Analysis:

Data Cleaning: Missing values are deleted and filled.

Normalization: Data are scaled to a range of [0, 1].

Feature Extraction: Using the Analytical Hierarchy Process (AHP) to assign weights and merge multimodal data sets.

Model Training: Features are trained using the R-CNN model, and classified using the SVM algorithm.

Experimental Results:

Grasping Success Rate and Cycle Comparison: Results are shown in Tables 3 and 4.

In 40 grasping tests, the proposed method maintained an average grasping success rate of 86.5% and an average cycle of 18.66 seconds across different scenarios.

### Method details

#### R-CNN model

The learning rate is set to 0.001, and an adaptive learning rate adjustment algorithm is used. This allows the learning rate to be adjusted dynamically during training, ensuring that the model learns quickly in the early stages and adjusts finely in the later stages. The batch size is set to 32. The number of training rounds is set to 100, and the performance of the validation set determines the optimal number of training rounds to prevent overfitting or underfitting. The momentum is set to 0.9. By introducing the momentum term, we are able to reach the optimal solution faster during training. The weight decay is 0.0005. It helps control the model complexity and ensures that the model does not remember the noise in the training set during training. The cross entropy loss is used as the loss function for the classification task. $L\left(y,\hat{y}\right)=-\sum_{i=1}^{n}y_{i}\;\log\left({\hat{y}}_{i}\right)$, where y is the true label and $\hat{y}$ is the predicted probability. The regression loss is used to adjust the regression of the bounding box, and the calculation formula is $L_{\text{reg}}\left(x,y\right)=\sum_{i=1}^{n}{\text{smoot}h}_{L1}\left(x_{i}-y_{i}\right)$, where ${\text{smoot}h}_{L1}$ is a loss function that linearizes larger errors and squares smaller errors.

#### Depth camera

In the experiment, using Kinect DK can make full use of its depth sensing and RGB image capture capabilities. Especially when dealing with transparent objects, Kinect DK's depth sensor can help reduce visual interference caused by transparency and reflection, thereby improving the accuracy of object recognition. The detailed parameters are as follows.

Depth Sensor:

Sensor Type: Time-of-Flight (ToF).

Resolution: 640 x 576 pixels Frame Rate: 5, 15, 30 FPS (configurable) Field of View (FOV): 75° x 65° (Horizontal x Vertical).

Depth Range:Indoor Mode: 0.5 m to 5.46 m

Outdoor Mode: 0.25 m to 2.88 m

RGB Camera:

Resolution: 3840 x 2160 pixels (4K) Frame Rate: 15, 30 FPS Field of View (FOV): 90° x 59° (Horizontal x Vertical) IMU (Inertial Measurement Unit): Gyroscope: ±2000 °/s Accelerometer: ±16 g

Connectivity and Interface: USB 3.0 Type-C

Power Supply: 12V DC adapter

This table provides a comprehensive overview of the Kinect DK’s capabilities, highlighting its depth sensing, RGB camera specifications, IMU parameters, and connectivity options.

#### Color dynamic recognition technology (CDR)

Color dynamic recognition technology (CDR) generates 3 feature images by fusing multimodal datasets, corresponding to brightness, transparency and color saturation respectively. The size of each feature image is 1×100×100 (channel×height×width).

#### Selective search algorithm

1) Each feature map (brightness, transparency, color saturation) is used as input, and the candidate region is generated by the Selective Search algorithm. Each feature map is processed separately to ensure the integrity of information under different modalities.
2) For each feature map, the Selective Search algorithm performs similarity calculation based on color similarity and region size similarity. The specific process is as follows:
  2.1) Initialize the candidate region set and equate the feature map to the initial candidate region.
  2.2) Calculate the color similarity metric and region size similarity metric.
  2.3) Select the region with the lowest similarity for segmentation and generate new candidate regions.
3) Select candidate regions from each feature map and perform weighted fusion on these regions. The specific allocation method is:
  3.1) Select 200 candidate regions from the brightness feature map. 00$\left(r_{1},r_{2},\cdots \cdots ,r_{2}\right)$.
  3.2) Select 150 candidate regions from the transparency feature map. 50$\left(r_{201},r_{202},\cdots \cdots ,r_{3}\right)$.
  3.3) Select 150 candidate regions from the color saturation feature map. 00$\left(r_{351},r_{352},\cdots \cdots ,r_{5}\right)$.
4) Weighted fusion of candidate regions extracted from each feature map. ${\text{Region}}_{\text{combined}}=w_{1}\cdot {\text{Region}}_{\text{Lum}}+w_{2}\cdot {\text{Region}}_{\text{Par}}+w_{3}\cdot {\text{Region}}_{\text{Col}}$.

${\text{Region}}_{\text{Lum}}$ represents the set of candidate regions of the brightness feature map. ${\text{Region}}_{\text{Par}}$ represents the set of candidate regions of the transparency feature map. ${\text{Region}}_{\text{Col}}$ represents the set of candidate regions of the color saturation feature map. The weights $w_{1},w_{2}$ and $w_{3}$ are determined by the analytic hierarchy process (AHP) to reflect the relative importance of each modality.

5) The final 500 weighted fused candidate regions are used as the input of the R-CNN model for further feature extraction and target recognition.

### Quantification and statistical analysis

The quantification and statistical analysis for this study were primarily conducted using MATLAB R2022a and Python. The detailed steps and methods are as follows:

Data Preprocessing

Statistical Analysis

1. Descriptive Statistics: The mean, median, standard deviation, and variance of the normalized datasets were calculated using MATLAB's built-in functions.

mean_Lum = mean(Lum_norm);

median_Lum = median(Lum_norm);

std_Lum = std(Lum_norm);

var_Lum = var(Lum_norm);

mean_Par = mean(Par_norm);

median_Par = median(Par_norm);

std_Par = std(Par_norm);

var_Par = var(Par_norm);

mean_Col = mean(Col_norm);

median_Col = median(Col_norm);

std_Col = std(Col_norm);

var_Col = var(Col_norm);

2. Correlation Analysis: Pearson correlation coefficients were calculated to determine the relationship between different features using the corrcoef function.

[R, P] = corrcoef([Lum_norm, Par_norm, Col_norm]);

3. Principal Component Analysis (PCA): PCA was performed to reduce the dimensionality of the data and identify the most significant features using the pca function.

[coeff, score, latent] = pca([Lum_norm, Par_norm, Col_norm]);

Grasping Success Rate Analysis

1. Success Rate Calculation: The grasping success rate for different scenarios was calculated as the ratio of successful grasps to total attempts.

success_rate = sum(successful_grasps) / total_grasps;

2. Cycle Time Analysis: The average cycle time for grasping in different scenarios was calculated.

average_cycle_time = mean(cycle_times);

Visualization

Plots and Graphs: Various plots were generated to visualize the data, including histograms, scatter plots, and PCA biplots.

figure;

histogram(Lum_norm);

title('Normalized Lum Values Histogram');

figure;

scatter3(Lum_norm, Par_norm, Col_norm);

title('3D Scatter Plot of Normalized Features');

figure;

biplot(coeff(:, 1:2), 'Scores', score(:, 1:2));

title('PCA Biplot');

## Supplemental information

Document S1. Tables S1–S3

Data S1. Butterfly optimization algorithm, depalletizing and palletizing structure source code, related to Figure 9
