# Supplementary material for: Color changing object recognition and grabbing technology based on crystal butterfly algorithm and adaptive imitation
Source: iScience. 2024 Jul 6;27(8):110457. doi: 10.1016/j.isci.2024.110457 (PMC11363504; doi:10.1016/j.isci.2024.110457)
Supplement: Document S1. Tables S1–S3 [file mmc1.pdf]

iScience, Volume *27*

Supplemental information

Color changing object recognition and grabbing technology based on crystal butterfly algorithm and adaptive imitation

Zuoxun Wang, Chuanyu Cui, Jinxue Sui, and Changkun Guo

Table S1: Initial data of transparent object attribute information before iteration, related to Figure 7.

Brightness	Transparency	Color saturation	Brightness	Transparency	Color saturation
3.402	2.442	1.8	2.33	1.997	1.392
3.381	2.433	1.792	2.309	1.988	1.384
3.36	2.424	1.784	2.288	1.98	1.376
3.338	2.415	1.776	2.267	1.971	1.368
3.317	2.407	1.768	2.246	1.962	1.36
3.296	2.398	1.76	2.225	1.953	1.352
3.275	2.389	1.752	2.204	1.945	1.344
3.254	2.381	1.744	2.183	1.936	1.336
3.233	2.372	1.736	2.162	1.927	1.328
3.212	2.363	1.728	2.141	1.919	1.32
3.191	2.354	1.72	2.12	1.91	1.312
3.17	2.346	1.712	2.099	1.902	1.304
3.149	2.337	1.704	2.078	1.893	1.296
3.128	2.328	1.696	2.057	1.884	1.288
3.107	2.32	1.688	2.036	1.877	1.28
3.086	2.311	1.68	2.015	1.868	1.272
3.065	2.302	1.672	1.994	1.86	1.264
3.044	2.293	1.664	1.973	1.853	1.256
3.023	2.285	1.656	1.952	1.844	1.248
3.002	2.276	1.648	1.931	1.837	1.24
2.981	2.267	1.64	1.91	1.828	1.232
2.96	2.258	1.632	1.889	1.82	1.224
2.939	2.25	1.624	1.868	1.812	1.216
2.918	2.241	1.616	1.847	1.803	1.208
2.897	2.232	1.608	1.826	1.795	1.2
2.876	2.224	1.6	1.805	1.787	1.192
2.855	2.215	1.592	1.784	1.779	1.184
2.834	2.206	1.584	1.763	1.771	1.176
2.813	2.197	1.576	1.742	1.761	1.168
2.792	2.189	1.568	1.721	1.751	1.16
2.771	2.18	1.56	1.7	1.742	1.152
2.75	2.171	1.552	1.679	1.734	1.144
2.729	2.163	1.544	1.658	1.721	1.136
2.708	2.154	1.536	1.637	1.719	1.128
2.687	2.145	1.528	1.616	1.706	1.12
2.666	2.136	1.52	1.595	1.696	1.112
2.645	2.128	1.512	1.574	1.692	1.104
2.624	2.119	1.504	1.553	1.681	1.096
2.603	2.11	1.496	1.532	1.669	1.088
2.582	2.102	1.488	1.511	1.665	1.08
2.561	2.093	1.48	1.49	1.647	1.072
2.54	2.084	1.472	1.469	1.639	1.064
2.519	2.075	1.464	1.448	1.629	1.056
2.498	2.067	1.456	1.427	1.625	1.048

2.477	2.058	1.448	1.406	1.611	1.04
2.456	2.049	1.44	1.385	1.602	1.032
2.435	2.041	1.432	1.364	1.591	1.024
2.414	2.032	1.424	1.343	1.577	1.016
2.393	2.023	1.416	1.322	1.562	1.008
2.372	2.015	1.408	1.301	1.551	1
2.351	2.006	1.4			

Table S2: Attribute data in Multi-modal Fusion Image Model training, related to Figure 4 and Figure 5.

Brightness	Transparency	Color saturation	Brightness	Transparency	Color saturation	Brightness	Transparency	Color saturation
0.403	0.119	0.918	0.375	0.122	0.863	0.383	0.130	0.846
0.976	0.467	0.665	0.951	0.495	0.623	0.917	0.518	0.613
0.799	0.028	0.344	0.732	0.034	0.331	0.751	0.037	0.328
0.608	1	0.065	0.599	0.909	0.064	0.570	0.978	0.066
0.16	0.272	0.315	0.156	0.259	0.311	0.150	0.276	0.302
0.171	0.607	0.361	0.156	0.663	0.325	0.160	0.621	0.346
0.053	0.288	0.763	0.058	0.312	0.730	0.053	0.296	0.729
0.936	0.461	0.696	0.866	0.520	0.638	0.873	0.513	0.664
0.672	0.517	0.95	0.601	0.547	0.887	0.625	0.574	0.904
0.761	0.163	0.487	0.708	0.185	0.472	0.708	0.189	0.464
0	1.024	0.092	0.021	0.970	0.120	0.020	0.994	0.120
0.982	0.735	0.741	0.970	0.775	0.713	0.977	0.800	0.706
0.815	1	0.805	0.832	0.939	0.761	0.811	0.971	0.774
0.196	0.95	0.583	0.212	0.895	0.561	0.205	0.927	0.561
0.168	0.636	0.789	0.182	0.598	0.771	0.176	0.621	0.758
0.183	0.999	0.51	0.183	0.922	0.494	0.189	0.970	0.492
0.299	0.074	0.545	0.304	0.088	0.523	0.310	0.084	0.526
0.494	0.177	0.456	0.525	0.196	0.428	0.516	0.205	0.430
0.408	0.032	0	0.432	0.045	0.025	0.424	0.043	0.028
0.27	0.292	0.086	0.291	0.325	0.108	0.285	0.320	0.106
0.672	0.35	0.024	0.612	0.389	0.031	0.626	0.384	0.034
0.125	0.243	0.682	0.139	0.271	0.636	0.133	0.267	0.623
0.298	0.797	0.34	0.292	0.829	0.314	0.309	0.839	0.312
0.391	0.331	0.548	0.366	0.357	0.509	0.379	0.356	0.501
0.499	0.261	0.948	0.456	0.281	0.908	0.472	0.282	0.903
0.81	0.509	0.186	0.785	0.543	0.249	0.795	0.549	0.247
0.189	0.13	0.375	0.200	0.141	0.410	0.196	0.144	0.404
0.483	0.78	0.773	0.514	0.802	0.756	0.507	0.822	0.741
0.592	0.069	0.203	0.592	0.075	0.229	0.605	0.077	0.230
0.025	1.056	0.057	0.046	0.987	0.077	0.049	1.032	0.077
0.571	0.754	0.275	0.608	0.772	0.290	0.589	0.806	0.289
0.173	0.179	0.153	0.171	0.199	0.161	0.163	0.207	0.162
0.039	0	0.969	0.065	0.006	0.930	0.068	0.005	0.910
0.969	0.796	0.857	0.949	0.815	0.808	0.944	0.837	0.809

0.971	0.692	0.666	0.966	0.707	0.633	0.946	0.728	0.613
0.816	0.719	0.923	0.808	0.729	0.871	0.812	0.757	0.857
0.31	0.745	0.86	0.305	0.771	0.804	0.320	0.781	0.798
0.104	0.07	0.169	0.098	0.074	0.187	0.106	0.078	0.195
0.712	0.341	0.973	0.684	0.358	0.893	0.695	0.376	0.913
0.461	0.115	0.573	0.440	0.116	0.539	0.456	0.122	0.547

Table S3: Butterfly trajectory tracking routes for various nodes during 5 depalletizing and palletizing gripping tests, related to Figure 3 and Figure 7.

X-axis	Y-axis	Z-axis	X-axis	Y-axis	Z-axis	X-axis	Y-axis	Z-axis	X-axis	Y-axis	Z-axis	X-axis	Y-axis	Z-axis
0.049	0.946	0.024	-0.043	1.087	0.017	0.052	1.046	0.007	0.062	1.036	0.017	0.084	1.045	0.001
0.207	1.008	0.353	0.283	0.979	0.351	0.254	1.01	0.34	0.264	1	0.34	0.244	0.998	0.334
0.542	0.931	0.676	0.529	0.878	0.678	0.577	0.885	0.674	0.577	0.895	0.678	0.561	0.888	0.674
0.822	0.732	1.011	0.833	0.74	1.015	0.811	0.676	1.004	0.821	0.676	1.004	0.803	0.678	1.002
1.039	0.487	1.35	1.062	0.502	1.356	1.098	0.455	1.339	1.088	0.445	1.339	1.093	0.452	1.338
0.913	0.185	1.672	1	0.214	1.681	0.982	0.181	1.676	0.972	0.171	1.676	0.975	0.191	1.675
1.17	-0.168	2.012	1.172	-0.159	2.023	1.201	-0.174	2.01	1.191	-0.164	2.01	1.179	-0.162	2.009
0.898	-0.509	2.344	0.923	-0.465	2.352	0.946	-0.46	2.345	0.936	-0.46	2.352	0.935	-0.459	2.348
0.629	-0.79	2.673	0.656	-0.785	2.673	0.642	-0.785	2.673	0.642	-0.785	2.673	0.654	-0.778	2.671
0.374	-0.968	3	0.391	-0.984	3.001	0.382	-0.967	3	0.382	-0.967	3	0.378	-0.967	2.998
-0.029	-1.104	3.326	-0.021	-1.076	3.325	-0.017	-1.072	3.326	-0.017	-1.072	3.326	-0.024	-1.075	3.326
-0.456	-0.921	3.654	-0.482	-0.937	3.654	-0.472	-0.915	3.654	-0.472	-0.915	3.654	-0.473	-0.923	3.652
-0.773	-0.756	3.981	-0.789	-0.731	3.981	-0.777	-0.725	3.981	-0.777	-0.725	3.981	-0.776	-0.726	3.98
-1.01	-0.516	4.309	-1.037	-0.51	4.308	-1.015	-0.508	4.309	-1.015	-0.508	4.309	-1.013	-0.507	4.309
-1.041	-0.151	4.633	-1.061	-0.122	4.635	-1.041	-0.125	4.633	-1.041	-0.125	4.633	-1.043	-0.135	4.635
-0.923	0.148	4.956	-0.924	0.19	4.956	-0.912	0.2	4.956	-0.912	0.2	4.956	-0.913	0.193	4.956
-0.724	0.421	5.282	-0.719	0.451	5.28	-0.711	0.455	5.282	-0.711	0.455	5.282	-0.713	0.453	5.281
-0.457	0.672	5.609	-0.471	0.676	5.609	-0.455	0.673	5.609	-0.455	0.673	5.609	-0.461	0.667	5.61
-0.159	0.856	5.937	-0.156	0.85	5.935	-0.16	0.855	5.937	-0.16	0.855	5.937	-0.153	0.851	5.935
0.149	0.96	6.263	0.143	0.955	6.264	0.15	0.97	6.263	0.15	0.97	6.263	0.143	0.956	6.263
0.447	0.981	6.59	0.452	0.968	6.59	0.447	0.984	6.59	0.447	0.984	6.59	0.442	0.973	6.59
0.728	0.897	6.916	0.734	0.903	6.916	0.738	0.902	6.916	0.738	0.902	6.916	0.733	0.896	6.915
0.981	0.738	7.243	0.993	0.735	7.243	0.995	0.738	7.243	0.995	0.738	7.243	0.994	0.735	7.243
1.192	0.512	7.57	1.198	0.524	7.57	1.2	0.512	7.57	1.2	0.512	7.57	1.199	0.514	7.57
1.346	0.257	7.896	1.346	0.246	7.896	1.346	0.267	7.896	1.346	0.267	7.896	1.347	0.268	7.896
1.438	-0.017	8.223	1.432	-0.032	8.223	1.435	-0.012	8.223	1.435	-0.012	8.223	1.436	-0.01	8.223
1.458	-0.312	8.55	1.459	-0.303	8.55	1.456	-0.302	8.55	1.456	-0.302	8.55	1.457	-0.304	8.55
1.4	-0.599	8.877	1.411	-0.598	8.877	1.404	-0.6	8.877	1.404	-0.6	8.877	1.403	-0.601	8.877
1.258	-0.866	9.203	1.267	-0.879	9.203	1.264	-0.863	9.203	1.264	-0.863	9.203	1.265	-0.862	9.203
1.038	-1.09	9.53	1.041	-1.092	9.53	1.043	-1.085	9.53	1.043	-1.085	9.53	1.043	-1.085	9.53
0.759	-1.26	9.856	0.745	-1.254	9.856	0.76	-1.25	9.856	0.76	-1.25	9.856	0.761	-1.252	9.856
0.433	-1.357	10.183	0.424	-1.348	10.183	0.423	-1.341	10.183	0.423	-1.341	10.183	0.425	-1.34	10.183
0.073	-1.372	10.51	0.065	-1.363	10.51	0.063	-1.361	10.51	0.063	-1.361	10.51	0.068	-1.359	10.51
-0.312	-1.299	10.836	-0.319	-1.308	10.836	-0.315	-1.3	10.836	-0.315	-1.3	10.836	-0.311	-1.299	10.836

-0.701	-1.14	11.163	-0.711	-1.143	11.163	-0.71	-1.13	11.163	-0.71	-1.13	11.163	-0.715	-1.133	11.163
-1.076	-0.911	11.489	-1.08	-0.9	11.489	-1.073	-0.905	11.489	-1.073	-0.905	11.489	-1.074	-0.906	11.489
-1.417	-0.616	11.816	-1.422	-0.615	11.816	-1.418	-0.6	11.816	-1.418	-0.6	11.816	-1.419	-0.599	11.816
-1.707	-0.265	12.143	-1.717	-0.264	12.143	-1.717	-0.262	12.143	-1.717	-0.262	12.143	-1.718	-0.262	12.143
-1.934	0.118	12.469	-1.931	0.128	12.469	-1.936	0.132	12.469	-1.936	0.132	12.469	-1.937	0.131	12.469
-2.085	0.516	12.796	-2.089	0.517	12.796	-2.089	0.508	12.796	-2.089	0.508	12.796	-2.088	0.506	12.796
-2.15	0.911	13.123	-2.15	0.907	13.123	-2.148	0.904	13.123	-2.148	0.904	13.123	-2.147	0.905	13.123
-2.123	1.288	13.449	-2.125	1.296	13.449	-2.128	1.295	13.449	-2.128	1.295	13.449	-2.126	1.295	13.449
-2.005	1.632	13.776	-2.006	1.639	13.776	-2.007	1.625	13.776	-2.007	1.625	13.776	-2.009	1.624	13.776
-1.796	1.929	14.102	-1.796	1.934	14.102	-1.798	1.923	14.102	-1.798	1.923	14.102	-1.798	1.923	14.102
-1.499	2.166	14.429	-1.5	2.168	14.429	-1.503	2.166	14.429	-1.503	2.166	14.429	-1.501	2.166	14.429
-1.122	2.33	14.756	-1.123	2.335	14.756	-1.125	2.328	14.756	-1.125	2.328	14.756	-1.125	2.328	14.756
-0.676	2.409	15.082	-0.679	2.397	15.082	-0.68	2.403	15.082	-0.68	2.403	15.082	-0.68	2.404	15.082
-0.173	2.392	15.409	-0.168	2.382	15.409	-0.168	2.387	15.409	-0.168	2.387	15.409	-0.168	2.386	15.409
0.355	2.272	15.735	0.357	2.272	15.735	0.358	2.267	15.735	0.358	2.267	15.735	0.359	2.266	15.735
0.889	2.042	16.062	0.882	2.042	16.062	0.886	2.035	16.062	0.886	2.035	16.062	0.886	2.034	16.062
1.407	1.704	16.389	1.405	1.71	16.389	1.405	1.713	16.389	1.405	1.713	16.389	1.405	1.714	16.389
1.887	1.267	16.715	1.887	1.267	16.715	1.887	1.262	16.715	1.887	1.262	16.715	1.888	1.261	16.715
2.311	0.748	17.042	2.313	0.757	17.042	2.313	0.757	17.042	2.313	0.757	17.042	2.312	0.757	17.042
2.661	0.168	17.368	2.651	0.16	17.368	2.658	0.163	17.368	2.658	0.163	17.368	2.659	0.165	17.368
2.925	-0.444	17.695	2.92	-0.432	17.695	2.927	-0.441	17.695	2.927	-0.441	17.695	2.927	-0.441	17.695
3.093	-1.068	18.022	3.085	-1.039	18.022	3.088	-1.036	18.022	3.088	-1.036	18.022	3.088	-1.036	18.022
3.16	-1.675	18.348	3.155	-1.648	18.348	3.153	-1.65	18.348	3.153	-1.65	18.348	3.152	-1.65	18.348
3.123	-2.235	18.675	3.129	-2.225	18.675	3.131	-2.222	18.675	3.131	-2.222	18.675	3.131	-2.222	18.675
2.983	-2.718	19.001	2.992	-2.702	19.001	2.988	-2.716	19.001	2.988	-2.716	19.001	2.988	-2.716	19.001
2.745	-3.09	19.328	2.754	-3.09	19.328	2.747	-3.092	19.328	2.747	-3.092	19.328	2.747	-3.092	19.328
